# Autophagic pathway is responsible for selective degradation of cytosolic enzymes formaldehyde and formate dehydrogenases in the methylotrophic yeast *Komagataella phaffii*

**DOI:** 10.1038/s41598-026-54529-6

**Published:** 2026-06-01

**Authors:** Olena V. Dmytruk, Uliana I. Pelypyshyn, Kostyantyn V. Dmytruk, Andriy A. Sibirny

**Affiliations:** 1 Institute of Cell Biology, National Academy of Sciences of Ukraine, Drahomanov St, 14/16, Lviv, 79005 Ukraine; 2 University of Rzeszow, Zelwerowicza 4, Rzeszow, 35-601 Poland

**Keywords:** Selective autophagy, ATG proteins, Vacuolar proteolysis, Protein turnover, Carbon source shift, Biochemistry, Cell biology, Microbiology, Molecular biology

## Abstract

In the methylotrophic yeast *Komagataella phaffii* (formerly *Pichia pastoris*), several methanol-induced enzymes are rapidly degraded upon a shift to glucose. We studied how deletion of autophagy-related genes affects turnover of two cytosolic enzymes, formaldehyde dehydrogenase (Fld1) and formate dehydrogenase (Fdh1), following the shift from methanol to glucose as a carbon source. GFP-tagged Fld1 and Fdh1 were expressed in wild-type and *atg1Δ*, *atg6Δ*, and *atg15Δ* strains, lacking, respectively, Atg1 (a serine/threonine kinase required for autophagosome formation), Atg6 (a subunit of phosphatidylinositol 3-kinase complexes I and II), and Atg15 (a phospholipase B required for lysis of autophagic bodies). Enzyme activities, Western blots, and fluorescence microscopy were used to monitor degradation. In wild-type cells Fld1 and Fdh1 levels and activities declined rapidly after the shift, whereas all *atg1Δ*, *atg6Δ* and *atg15Δ* mutants retained substantially higher Fld1/Fdh1 activity, similar to the peroxisomal enzyme alcohol oxidase, and cytosolic GFP signal. These data show that Atg1, Atg6 and Atg15 are required for efficient vacuolar degradation of cytosolic enzymes of methanol metabolism Fld1 and Fdh1. We conclude that these cytosolic enzymes are cleared via selective autophagy upon cell shift from methanol to glucose, underscoring the essential role of the autophagy–lysosome pathway in proteome remodeling of *K. phaffii*.

**Supplementary Information:**

The online version contains supplementary material available at 10.1038/s41598-026-54529-6.

## Introduction

Protein homeostasis in eukaryotic cells depends on a tightly regulated balance between synthesis and degradation^1^. Two major degradative pathways maintain this balance: the ubiquitin–proteasome system (UPS) and the autophagy–lysosome/vacuole pathway^2^. Generally, the proteasome preferentially degrades short-lived regulatory proteins and misfolded peptides, whereas autophagy engulfs long-lived proteins, protein aggregates, and even entire organelles for degradation in the vacuole^3^. Autophagy is an evolutionarily conserved cellular process through which cytoplasmic components, including proteins and organelles, are delivered to the lysosome or vacuole in yeast for degradation and recycling^4^. Autophagy-related (*ATG*) genes encode proteins essential for autophagy. Autophagy can be divided into two main types, microautophagy and macroautophagy, and both of these include nonselective and selective processes^5^. Microautophagy and macroautophagy differ primarily in how cytoplasmic material is delivered to the lysosome: in microautophagy the lysosomal membrane directly engulfs cargo by invagination or protrusion, whereas in macroautophagy cytoplasmic constituents are sequestered within a newly formed double-membrane autophagosome that subsequently fuses with the lysosome for degradation. Furthermore, both microautophagy and macroautophagy can operate in a nonselective mode, characterized by bulk degradation of random regions of the cytoplasm under conditions such as starvation, or in a selective mode, in which specific substrates, including damaged or no longer needed organelles, are targeted for lysosomal degradation through dedicated recognition mechanisms and receptor proteins^6^. In selective macroautophagy, cargo sequestration is mediated by specific receptor proteins (e.g., Atg19 for cytoplasm-to-vacuole targeting pathway, Atg32 for mitophagy, and Atg36 in *Saccharomyces cerevisiae* or Atg30 in *Komagataella phaffii* (formerly *Pichia pastoris*) for pexophagy) that recognize target substrates and bind to both Atg8 and scaffold protein Atg11, ensuring selective incorporation into autophagosomes. Autophagosome formation is initiated by the Atg1 kinase complex at the phagophore assembly site, followed by the phosphatidylinositol 3-kinase complex (containing Atg6) that generates phosphatidylinositol 3-phosphate for membrane nucleation; receptor interactions with Atg8/Atg11 are often regulated sequentially by phosphorylation. Expansion and closure involve Atg8 lipidation and other Atg proteins for membrane dynamics. The completed autophagosome fuses with the vacuole, where inner autophagic bodies are lysed by hydrolases including lipase Atg15, enabling cargo degradation and macromolecule recycling^6^.

In yeast, changes in carbon source, particularly a shift from non-fermentable substrates to glucose, trigger the selective autophagic degradation of specific metabolic enzymes, a process known as catabolite inactivation. In yeast, shifts in carbon source are accompanied by extensive transcriptional and metabolic reprogramming. In methylotrophic yeasts such as *K. phaffii* , transfer of cells from methanol to glucose results in rapid catabolite repression of genes involved in methanol utilization and peroxisome biogenesis, while genes associated with glucose uptake and glycolysis become strongly induced^7^. These global changes in gene expression enable the cell to switch from methanol-based metabolism to glucose-supported growth. In addition to transcriptional reprogramming, metabolic adaptation requires the elimination of previously synthesized enzymes that become redundant following a shift in the carbon source. A key process contributing to this turnover is autophagy-mediated degradation of metabolic enzymes. This pathway facilitates efficient metabolic reprogramming by targeting redundant enzymes, such as those involved in gluconeogenesis, for delivery to the vacuole via the core autophagy machinery. In *S. cerevisiae*, key gluconeogenic enzymes such as fructose-1,6-bisphosphatase (Fbp1), isocitrate lyase (Icl1), and phosphoenolpyruvate carboxykinase (Pck1) are rapidly inactivated and degraded upon glucose addition via selective autophagy pathways that involve sequestration into specialized vesicles and subsequent vacuolar degradation^6,8^. Switching *S. cerevisiae* from glucose to non-fermentable carbon sources (such as ethanol) rapidly induces autophagy, driving the turnover of cytosolic proteins and facilitating adaptation to respiratory growth^9^.

Methylotrophic yeasts exhibit a distinct response to carbon source shifts, whereby in *K. phaffii*, enzymes of methanol assimilation induced during growth on methanol are rapidly degraded upon transfer to glucose or ethanol^10^. For instance, cytosolic formate dehydrogenase (Fdh1) and peroxisomal alcohol oxidase (Aox1) are synthesized on methanol but become targets of autophagic degradation upon glucose adaptation^10^.

*K. phaffii* has emerged as a powerful model organism for fundamental cell biology. Phylogenetically distant from *S. cerevisiae* and retaining many ancestral traits, *K. phaffii* is obligately aerobic and methylotrophic, efficiently assimilating methanol as its sole carbon and energy source^11^. This unique metabolism has made it a focus of research on peroxisome biogenesis and pexophagy (selective peroxisome autophagy). Indeed, multiple *ATG* genes and related regulators mediating pexophagy have been identified in *K. phaffii* and related yeasts^12^. Mutants in these genes show defective peroxisome turnover, underscoring the well-characterized pathways for organelle degradation in methylotrophic yeasts. By contrast, much less is known about the degradation of endogenous cytosolic enzymes in *K. phaffii*. Aside from these organelle-specific studies, only a few cytosolic targets have been described. For example, Fbp1 is known to be delivered to the vacuole via autophagy in *K. phaffii* under glucose adaptation^13^. Similarly, the glucose-induced degradation of cytosolic Fdh1 has been noted^10^, but the general mechanisms targeting cytosolic proteins for autophagy remain largely unexplored.

Here, we investigate the degradation of two key cytosolic enzymes of methanol catabolism, formaldehyde dehydrogenase (Fld1) and Fdh1, in *K. phaffii*. Using strains expressing tagged Fld1 and Fdh1, we monitored enzyme activity, performed Western blot analysis, and applied fluorescence microscopy in cells grown on methanol and then shifted to glucose. These experiments clarify the autophagic versus proteasomal contributions to Fld1 and Fdh1 turnover and illuminate the mechanisms by which *K. phaffii* degrades its cytosolic methanol-metabolism enzymes.

## Results

### Pathways of degradation Fld1 and Fdh1

**Fig. 1:**
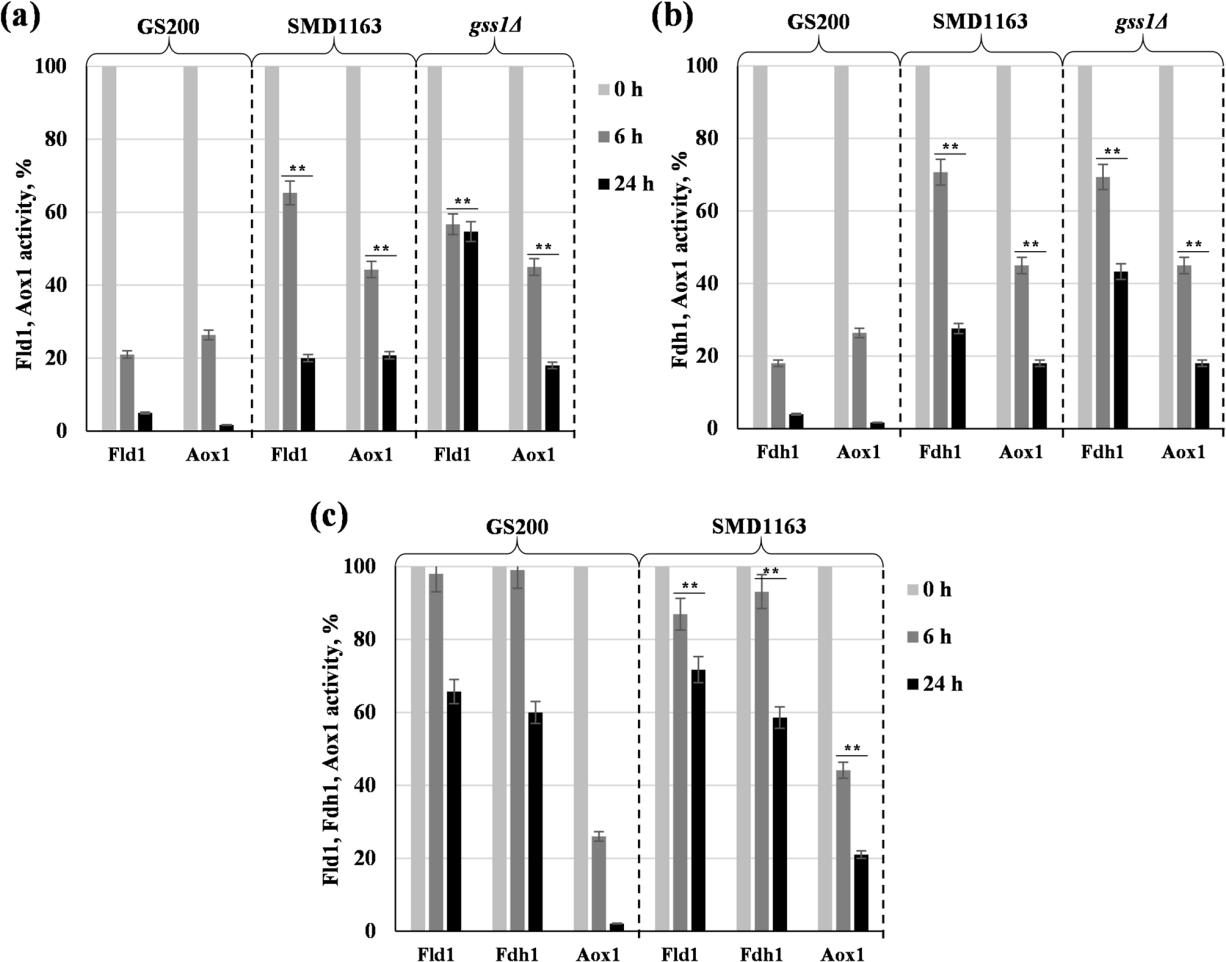
Relative activity (%) of Fld1 (**a**), Fdh1 (**b**) and Aox1 on 0, 6, 24 h after the transfer of cells from methanol to glucose-containing medium in *K. phaffii* strains GS200, SMD1163 and *gss1Δ*; (**с**) relative activity (%) of Fld1, Fdh1 and Aox1 on 0, 6, 24 h after the transfer of cells from methanol to ethanol-containing medium in strains GS200 and SMD1163. Data are presented as mean ± SD (*n* = 3 biological replicates). Differences relative to the GS200 strain at the corresponding time point are indicated as *p* < 0.01 (**), Student’s *t*-test.

Fld1 and Fdh1 are valuable model proteins for studying cytosolic protein degradation in methylotrophic yeast, as they play key roles in methanol metabolism. In this process, methanol is oxidized to formaldehyde by Aox1, which is then converted to formate by Fld1, and finally, Fdh1 catalyzes the oxidation of formate to carbon dioxide. Specific activity of Fld1 and Fdh1 rapidly declines upon shifting methylotrophic-grown cells to a glucose medium. We investigated the inactivation of Fld1 and Fdh1 in the wild-type strain *K. phaffii* GS200 and strains deficient in vacuolar proteases (SMD1163) and impaired glucose sensing (*gss1Δ*). In SMD1163, reduced vacuolar protease activity impairs the final degradation step of autophagic bodies after vacuolar fusion, leading to the accumulation of undegraded cargo and prolonged retention of peroxisomal proteins despite normal initiation of pexophagy^10^. The *gss1Δ* strain lacks the glucose sensor Gss1, which is involved in glucose catabolite repression and contributes to the regulation of autophagic degradation of peroxisomes in *K. phaffii*^14^. The specific activity of Fld1 and Fdh1 in strains SMD1163 and *gss1Δ* remained at least twofold higher compared to the activity of Fld1 and Fdh1 in the wild-type strain after shifting cells from methanol to glucose (Fig. 1a, b), suggesting a vacuolar degradation pathway for these enzymes. A similar trend with a more pronounced difference was observed for the model peroxisomal enzyme Aox1, where activity in the mutants remained about tenfold higher than in the wild-type after shift from methanol to glucose (Fig. 1a, b). Aox1 degradation has been reported to occur via the autophagic pathway^15^. The specific activities of Fld1 and Fdh1 were also measured in the GS200 and SMD1163 strains following a shift from methanol to ethanol. In contrast to glucose adaptation, ethanol adaptation did not alter Fld1 or Fdh1 activities within 6 h after the shift (Fig. 1c). In comparison, Aox1 activity in GS200 decreased rapidly, similarly to the decline observed after adaptation to glucose. A retardation of Aox1 activity was observed in the SMD1163 strain during both ethanol and glucose adaptation (Fig. 1), consistent with previous observations reported by Tuttle and Dunn^10^.

**Fig. 2:**
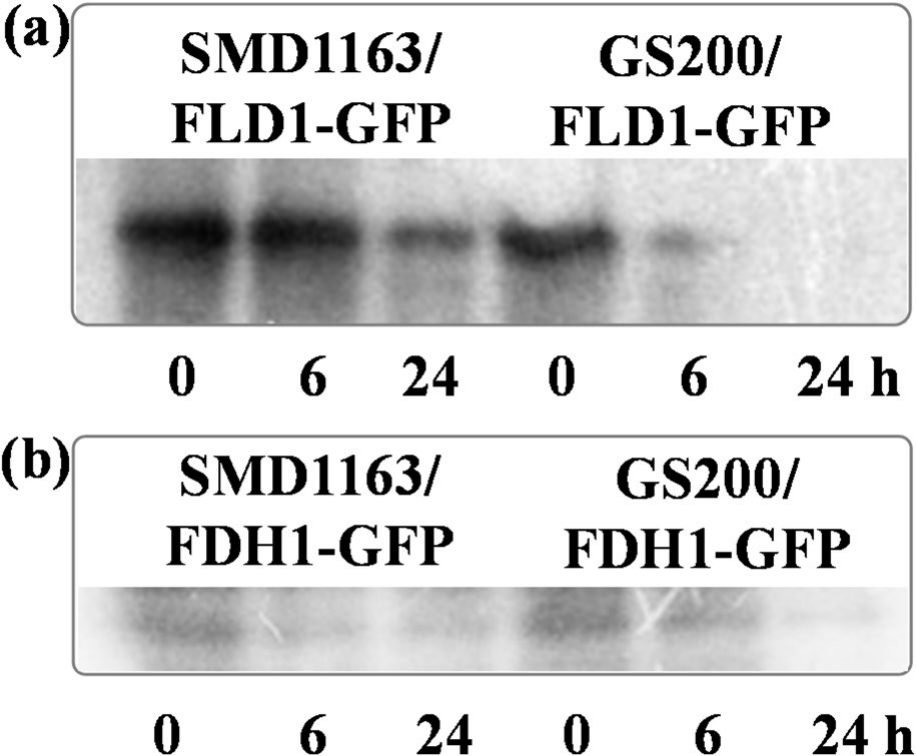
Degradation of (**a**) Fld1 and (**b**) Fdh1 following the transfer of cells from methanol to glucose-containing medium for 0, 6, 24 h in *K. phaffii* strains GS200/FLD1-GFP, SMD1163/FLD1-GFP, GS200/FDH1-GFP, and SMD1163/FDH1-GFP. Anti-GFP antibody was used for Western blot analysis. Uncropped Western blot and corresponding membrane shown in Fig. S1 and Fig. S2, respectively.

To validate the conclusions drawn from the activity assays of the Fld1 and Fdh1 enzymes, strains expressing the target enzymes fused with GFP were constructed as outlined in the Materials and Methods section. The degradation of Fld1 and Fdh1 was then evaluated through both Western blotting and fluorescence microscopy in the strains GS200/FLD1-GFP, SMD1163/FLD1-GFP, GS200/FDH1-GFP, and SMD1163/FDH1-GFP. Based on the results of Western blot analysis, it was observed that Fld1 and Fdh1 undergo complete degradation on 24 h after shifting GS200/FLD1-GFP and GS200/FDH1-GFP cells from methanol to glucose (Fig. 2). However, the degradation of Fld1 and Fdh1 is impaired in SMD1163/FLD1-GFP and SMD1163/FDH1-GFP due to the block of vacuolar proteases, indicating that the degradation of Fld1 and Fdh1 occurs through the vacuolar pathway.

**Fig. 3:**
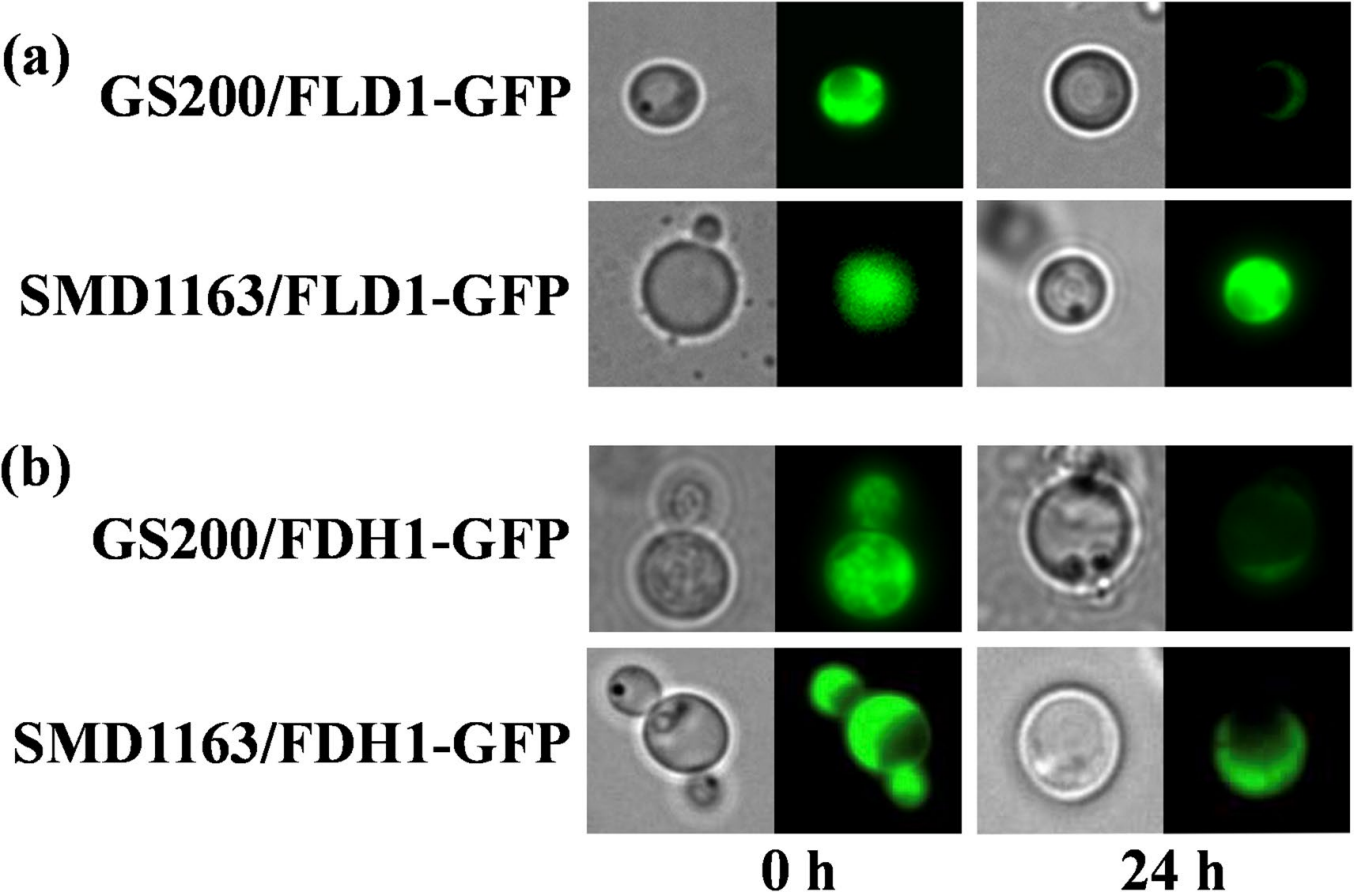
Fluorescence images of the GS200/FLD1-GFP, SMD1163/FLD1-GFP (**a**), GS200/FDH1-GFP and SMD1163/FDH1-GFP (**b**) strains, which were grown on methanol medium and then transferred to glucose medium for 24 h to stimulate the degradation of Fld1 and Fdh1 in *K. phaffii*.

To visualize the degradation of Fld1 and Fdh1, the cells of strains GS200/FLD1-GFP, SMD1163/FLD1-GFP, GS200/FDH1-GFP, and SMD1163/FDH1-GFP were observed under fluorescence microscope. After incubation in methanol medium, cells from all strains exhibited a dispersed green glow in the cytoplasm, confirming the cytosolic localization of Fld1-GFP and Fdh1-GFP fusions (Fig. 3). GFP fluorescence is disappeared on 24 h after shifting GS200/FLD1-GFP and GS200/FDH1-GFP cells from methanol to glucose. At the same time, fluorescence is retained in strains SMD1163/FLD1-GFP and SMD1163/FDH1-GFP after their shifting to glucose (Fig. 3). These findings were further supported by fluorescence measurements in cell suspensions, which revealed significantly higher GFP signal in SMD1163/FLD1-GFP and SMD1163/FDH1-GFP strains compared to GS200/FLD1-GFP and GS200/FDH1-GFP after the shift to glucose. Specifically, fluorescence intensity was increased by 5.7-fold and 5.5-fold, respectively (Fig. S3). The obtained results confirm the vacuolar degradation of Fld1 and Fdh1, which are consistent with the results of the Western blot and the enzyme activity assays.

### Impact of *atg1Δ*, *atg6Δ*, and *atg15Δ* on the degradation of Fld1 and Fdh1

Comprehensive research has been conducted on the mechanisms of yeast autophagy^6^. The molecular machinery of autophagy, along with the genes essential for this process, has been identified^16^. It was intriguing to explore the effects of disrupting genes associated with different stages of autophagy on the degradation of Fld1 and Fdh1. Genes *ATG1* (essential for autophagy initiation and vesicle formation), *ATG6* (involved in autophagosome formation), and *ATG15* (associated with the disruption of autophagic bodies in the vacuole) were selected. Genes *ATG1*, *ATG6*, and *ATG15* were deleted on the background of GS200/FLD1-GFP and GS200/FDH1-GFP, resulting in the construction of strains GS200/FLD1-GFP/*atg1Δ*, GS200/FLD1-GFP/*atg6Δ*, GS200/FLD1-GFP/*atg15Δ*, GS200/FDH1-GFP/*atg1Δ*, GS200/FDH1-GFP/*atg6Δ*, GS200/FDH1-GFP/*atg15Δ*, as described in Materials and Methods.

A common phenotype of autophagy-deficient mutants is reduced viability during incubation in starvation conditions. Therefore, the viability of the constructed *atg* mutants was evaluated under nitrogen starvation, which induces general autophagy, using the Phloxine B assay. Phloxine B, a red dye and fluorescein derivative, is excluded from living yeast cells but penetrates and stains cytosolic components once the cell dies and the plasma membrane integrity is compromised^17^. The parental strains GS200/FLD1-GFP and GS200/FDH1-GFP were used as positive controls, whereas the SMD1163 strain, which is defective in vacuolar proteinases, was used as the negative control. After one day of nitrogen starvation, fluorescence analysis of Phloxine B treated cells revealed that general autophagy was impaired in all *atg* mutants. The fluorescence intensity of *atg6Δ* was comparable to that of SMD1163, whereas *atg1Δ* and *atg15Δ* exhibited approximately threefold lower fluorescence than SMD1163 and threefold higher than GS200 (Fig. S4a). Hence, we conclude that general autophagy is impaired in *atg1Δ* and *atg15Δ*, and more strongly in *atg6Δ*, which is consistent with previously reported data for *atg1Δ*^18^. Fluorescence microscopy further supported these findings, showing the strongest impairment of autophagy in *atg6Δ*, and milder defects in *atg1Δ* and *atg15Δ* compared with the control strains (Fig. S4). Nitrogen starvation resulted in nearly 100% of stained cell in the *atg6Δ* strains, similar to SMD1163 (Fig. S4b).

**Fig. 4:**
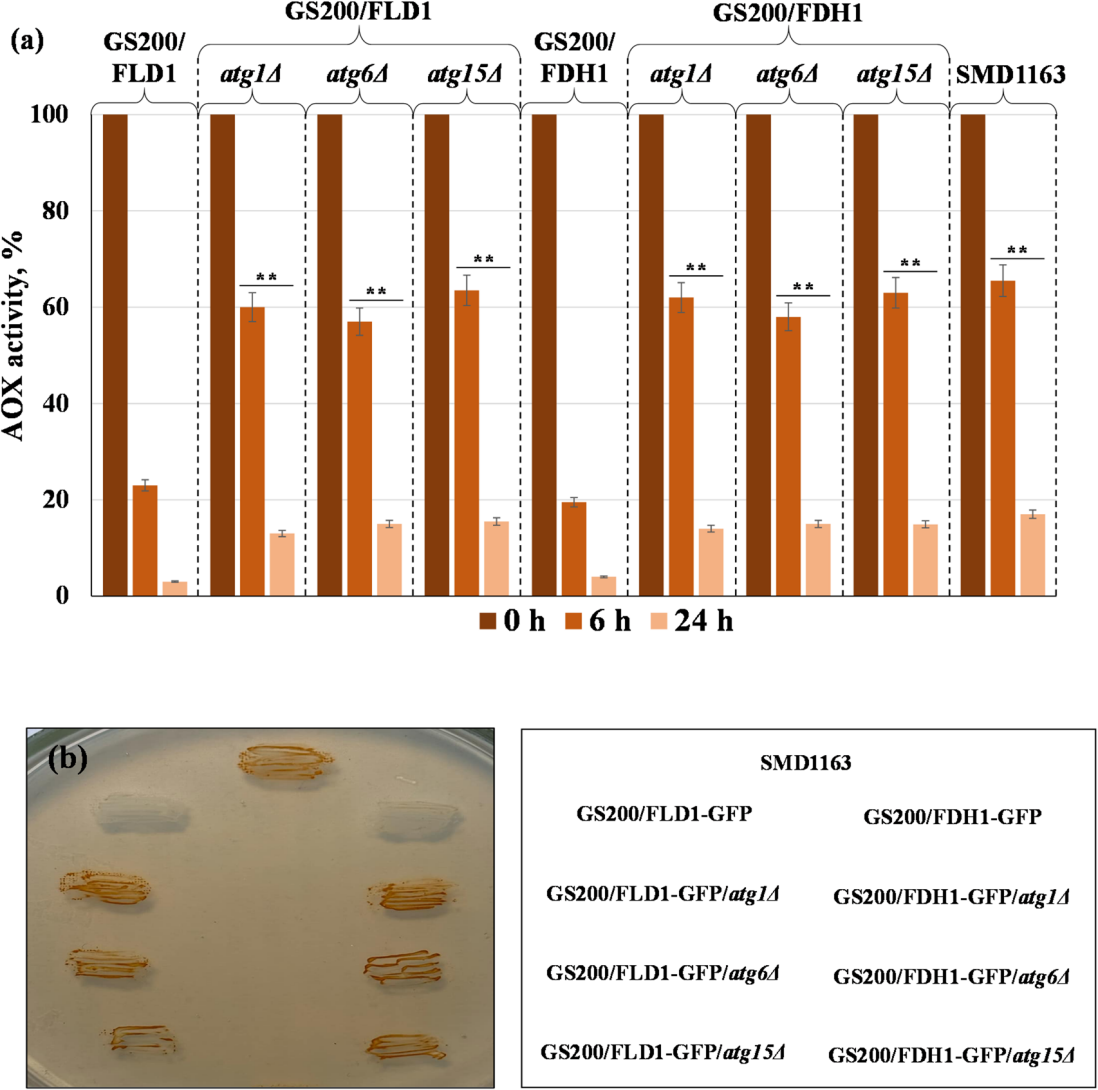
(**a**) Specific activity of AOX on 0, 6, 24 h after the transfer of cells from methanol to glucose-containing medium in strains GS200/FLD1-GFP, GS200/FLD1-GFP/*atg1Δ*, GS200/FLD1-GFP/*atg6Δ*, GS200/FLD1-GFP/*atg15Δ*; GS200/FDH1-GFP, GS200/FDH1-GFP/*atg1Δ*; GS200/FDH1-GFP/*atg6Δ*, GS200/FDH1-GFP/*atg15Δ* and SMD1163. Data are presented as mean ± SD (*n* = 3 biological replicates). Differences relative to the GS200/FLD1-GFP or GS200/FDH1-GFP strains at the corresponding time point are indicated as *p* < 0.01 (**), Student’s *t*-test. (**b**) Visualization of AOX activity after 8 h of glucose adaptation in parental strains GS200/FLD1-GFP, GS200/FDH1-GFP, corresponding *atg* mutants and SMD1163.

An Aox1 assay in cell free extracts was used to study micropexophagy in the constructed mutants *atg1Δ*, *atg6Δ*, and *atg15Δ*. *K. phaffii* GS200/FLD1-GFP, GS200/FDH1-GFP, SMD1163, GS200/FLD1-GFP/*atg1Δ*, GS200/FDH1-GFP/*atg1Δ*, GS200/FLD1-GFP/*atg6Δ*, GS200/FDH1-GFP/*atg6Δ*, GS200/FLD1-GFP/*atg15Δ*, and GS200/FDH1-GFP/*atg15Δ* strains were cultured on methanol minimal medium for 24 h, followed by shift in glucose minimal medium to induce micropexophagy. Mutants *atg1Δ*, *atg6Δ*, and *atg15Δ* exhibited similar residual Aox1 activity, reaching 57.0–63.5% after 6 h of incubation on glucose, comparable to SMD1163, which has a blocked pexophagy pathway. In contrast, GS200/FLD1-GFP and GS200/FDH1-GFP showed only approximately 20% Aox1 activity under the same conditions (Fig. 4a). The defect in Aox1 degradation in *atg1Δ*, *atg6Δ*, and *atg15Δ* was further confirmed by a plate patch assay on glucose-containing solid medium after pregrowth on methanol (Fig. 4b). The obtained results showing that disruption of the *ATG1*, *ATG6*, and *ATG15* genes leads to a defect in Aox1 degradation are consistent with previously reported findings for *ATG1* and *ATG6*^19^.

The degradation of Fld1 and Fdh1 was investigated in strains GS200/FLD1-GFP/*atg1Δ*, GS200/FLD1-GFP/*atg6Δ*, GS200/FLD1-GFP/*atg15Δ*, GS200/FDH1-GFP/*atg1Δ*, GS200/FDH1-GFP/*atg6Δ*, GS200/FDH1-GFP/*atg15Δ* through the assessment of specific enzymatic activity, Western blot analysis, and fluorescent microscopy.

**Fig. 5:**
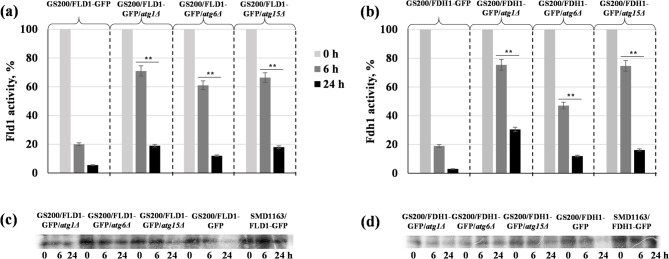
(**a**) Specific activity of Fld1 on 0, 6, 24 h after the transfer of cells from methanol to glucose-containing medium in strains GS200/FLD1-GFP, GS200/FLD1-GFP/*atg1Δ*, GS200/FLD1-GFP/*atg6Δ*, GS200/FLD1-GFP/*atg15Δ*. Data are presented as mean ± SD (*n* = 3 biological replicates). Differences relative to the GS200/FLD1-GFP strain at the corresponding time point are indicated as *p* < 0.01 (**), Student’s *t*-test. (**b**) Specific activity of Fdh1 under the same conditions in strains GS200/FDH1-GFP, GS200/FDH1-GFP/*atg1Δ*, GS200/FDH1-GFP/*atg6Δ*, GS200/FDH1-GFP/*atg15Δ*. Data are presented as mean ± SD (*n* = 3 biological replicates). Differences relative to the GS200/FDH1-GFP strain at the corresponding time point are indicated as *p* < 0.01 (**), Student’s *t*-test. (**c**) Degradation of Fld1 following the transfer of cells from methanol to glucose-containing medium for 0, 6, 24 h in *K. phaffii* strains GS200/FLD1-GFP/*atg1Δ*, GS200/FLD1-GFP/*atg6Δ*, GS200/FLD1-GFP/*atg15Δ* and control strains GS200/FLD1-GFP and SMD1163-FLD1-GFP. Uncropped Western blot and corresponding membrane shown in Fig. S5. (**d**) Degradation of Fdh1 under the same conditions in strains GS200/FDH1-GFP, GS200/FDH1-GFP/*atg1Δ*, GS200/FDH1-GFP/*atg6Δ*, GS200/FDH1-GFP/*atg15Δ* and control strains GS200/FDH1-GFP and SMD1163-FDH1-GFP. Uncropped Western blot and corresponding membrane shown in Fig. S6. Anti-GFP antibody was used for Western blot analysis.

The specific activity of Fld1 and Fdh1 in the *atg1Δ*, *atg6Δ*, and *atg15Δ* strains was measured under the conditions of methanol induction with subsequent shifting to glucose to induce the degradation of target enzymes. It was observed that the activities of Fld1 and Fdh1 in the strains GS200/FLD1-GFP/*atg1Δ*, GS200/FLD1-GFP/*atg6Δ*, GS200/FLD1-GFP/*atg15Δ*, as well as GS200/FDH1-GFP/*atg1Δ*, GS200/FDH1-GFP/*atg6Δ*, and GS200/FDH1-GFP/*atg15Δ*, remained high after 6 h of cultivation on glucose, reaching 61–71% and 47–75%, respectively, compared to the corresponding parental strains, in which enzyme activities decreased to 20% and 19%, respectively (Fig. 5a, b). The relatively high activities of Fld1 and Fdh1 in *ATG* deletion strains upon transfer to glucose medium mirrored the pattern observed in SMD1163 (Fig. 1). Notably, the enzymatic activities of Fld1 and Fdh1 in strains expressing GFP-tagged proteins were comparable to those observed in the parental GS200 strain expressing native, untagged enzymes (Fig. 1), suggesting that GFP fusion does not significantly affect the stability or degradation behavior of these proteins under the conditions tested. Hence, deletion of *ATG1*, *ATG6*, and *ATG15* slows down Fld1 and Fdh1 degradation. The results of Western blot analysis indicated that deletion of *ATG1*, *ATG6*, and *ATG15* limited the degradation of Fld1 and Fdh1 (Fig. 5c, d), which is consistent with the results of the specific enzyme activity assays.

**Fig. 6:**
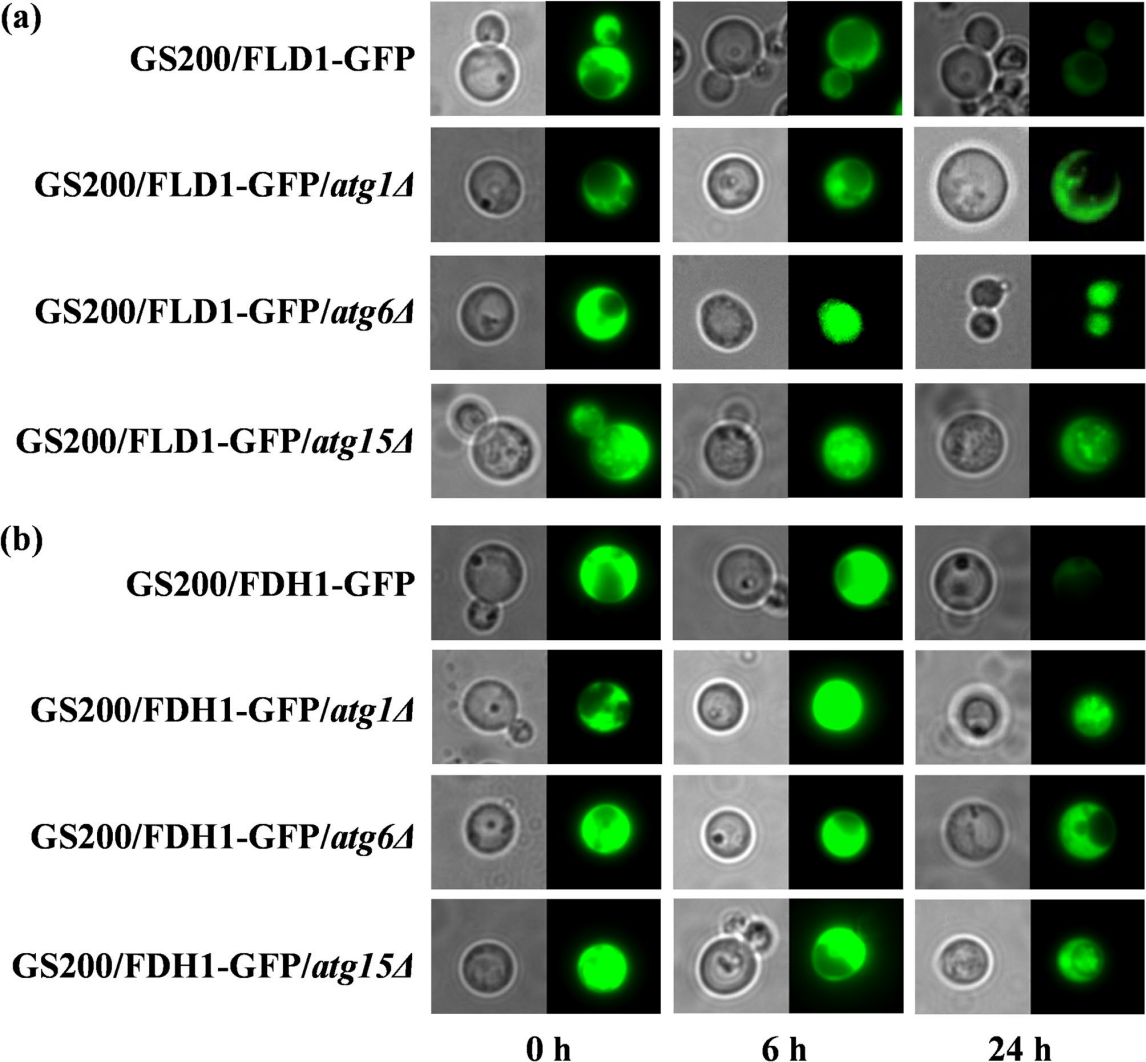
Fluorescence images of the (**a**) GS200/FLD1-GFP, GS200/FLD1-GFP/*atg1Δ*, GS200/FLD1-GFP/*atg6Δ*, GS200/FLD1-GFP/*atg15Δ*, and (**b**) GS200/FDH1-GFP, GS200/FDH1-GFP/*atg1Δ*, GS200/FDH1-GFP/*atg6Δ*, GS200/FDH1-GFP/*atg15Δ* strains, which were grown on methanol medium and then transferred to glucose medium to stimulate the degradation of Fld1 and Fdh1 in *K. phaffii*.

To validate the results of the specific activity of Fld1 and Fdh1, and Western blot analysis, the *atg1Δ*, *atg6Δ*, and *atg15Δ* mutants of *K. phaffii* were examined using fluorescence microscopy. As shown in Fig. 6, the GS200/FLD1-GFP/*atg1Δ*, GS200/FLD1-GFP/*atg6Δ*, GS200/FLD1-GFP/*atg15Δ*, GS200/FDH1-GFP/*atg1Δ*, GS200/FDH1-GFP/*atg6Δ* and GS200/FDH1-GFP/*atg15Δ* strains, unlike the parental strains, exhibited GFP fluorescence at 24 h after the shift from methanol to glucose, confirming the important role of Atg1, Atg6, and Atg15 in the autophagic degradation of Fld1 and Fdh1. Fluorescence measurements in cell suspensions further supported these observations, revealing a 5- to 6-fold increase in GFP signal in the deletion strains compared to the corresponding parental controls after the shift to glucose, consistent with the microscopy data (Fig. S3).

We also examined biomass accumulation of the *atg1Δ*, *atg6Δ*, and *atg15Δ* strains on both glucose and methanol. For this purpose, the strains were pre-cultured in YPD liquid medium for 24 h and subsequently inoculated into YNB medium containing 2% glucose or 1% methanol as the carbon source (Fig. S7). The *atg1Δ* and *atg15Δ* strains exhibited approximately 13% lower biomass accumulation, reaching about 5 OD after 72 h of growth in liquid mineral medium containing glucose compared to the parental strains (Fig. S7a, S7c). In contrast, the *atg6Δ* strain showed a more pronounced reduction in biomass, reaching about 2 OD after 72 h and decreasing by 2.8 fold in glucose medium compared to the parental strains (Fig. S7a, S7c). A similar trend was observed in mineral medium containing methanol. The *atg1Δ* and *atg15Δ* strains accumulated about 32% less biomass, reaching approximately 2 OD after 72 h of cultivation compared to the parental strains (Fig. S7b, S7d). The *atg6Δ* strain exhibited a pronounced growth defect, with biomass reaching only 0.8 OD after 72 h, corresponding to a 3.3-fold decrease relative to the parental strains (Fig. S7b, S7d). The increased fluorescence observed in the *atg6Δ* strain (Fig. S4) reflects reduced cell viability and is consistent with its pronounced defect in biomass accumulation observed in growth experiments.

## Discussion

In *K. phaffii*, shifts in the carbon source elicit distinct autophagic pathways. Specifically, adaptation cells from methanol to glucose induces microautophagy, predominantly micropexophagy, during which the vacuolar membrane invaginates or forms finger-like extensions that directly engulf peroxisomes and sequester them into the vacuolar lumen for degradation. In contrast, shifting cells from methanol to ethanol triggers macroautophagy, specifically macropexophagy, whereby individual peroxisomes are first enclosed within double-membrane vesicles known as autophagosomes, originating from the pre-autophagosomal structure (PAS), which subsequently fuse with the vacuole^6^. In this context, based on measurements of Fld1 and Fdh1 activities during adaptation to ethanol and glucose, we conclude that these enzymes are degraded via microautophagy. Our results are in good agreement with previous findings obtained from studies of Fdh1 degradation during adaptation of *K. phaffii* cells to ethanol and glucose^10^. At present, the precise mechanism underlying the recognition and sequestration of Fld1 and Fdh1 during glucose adaptation and microautophagy remains unclear and can only be hypothesized. Possible mechanisms include the concentration of Fld1 and Fdh1 in the vicinity of peroxisomes, leading to their co-engulfment with peroxisomes; some form of specific recognition by the vacuole; or interaction with a carrier protein followed by direct delivery into the vacuole.

Our results demonstrate that deletion of *ATG1*, *ATG6*, and *ATG15* in *K. phaffii* substantially affects the degradation dynamics of the cytosolic enzymes Fld1 and Fdh1 upon the metabolic shift from methanol to glucose. The impairment of Fld1 and Fdh1 degradation in the *atg* mutants, confirmed by both enzymatic activity assays and fluorescence microscopy, clearly indicates that these core *ATG* genes are essential for maintaining autophagic turnover of redundant enzymes during carbon source transition selectively to glucose but not to ethanol medium. This finding aligns with previously reported roles of *ATG1* and *ATG6* in autophagy initiation and phagophore formation, as well as *ATG15* in vacuolar degradation of autophagic bodies^19,20^. Our results further indicate that disruption of the *ATG1*, *ATG6*, and *ATG15* genes impairs micropexophagy, as evidenced by defective Aox1 degradation. Thus, the same *ATG* genes are involved in both selective pexophagy and selective autophagy of cytosolic proteins. Importantly, unlike Aox1, which is localized in peroxisomes and therefore represents a canonical substrate of micropexophagy, both Fld1 and Fdh1 are cytosolic enzymes. This localization argues against their degradation through pexophagic pathways that selectively target peroxisomes. Instead, the observed turnover of Fld1 and Fdh1 during glucose adaptation is more consistent with selective microautophagic degradation of cytosolic proteins. In line with the mechanistic features of microautophagy, the vacuole engulfs portions of the cytoplasm or entire organelles rather than individual soluble proteins; therefore, Fld1 and Fdh1 are unlikely to be substrates of micropexophagy. Notably, the observed degradation pattern does not appear to reflect nonspecific bulk autophagy, as the turnover specifically affects methanol-metabolizing enzymes during the metabolic shift from methanol to glucose, rather than indicating generalized degradation of cytosolic proteins.

The observed reductions in biomass accumulation for the *atg1Δ*, *atg6Δ*, and *atg15Δ* strains on both glucose and methanol likely reflect general impairments in autophagy-related processes. These mutants exhibit decreased cellular fitness, consistent with the essential housekeeping roles of the corresponding *ATG* genes. Consistent with these findings, data from the *Saccharomyces* Genome Database (https://frontend.qa.yeastgenome.org/) indicate slower growth kinetics for the corresponding mutants. The pronounced growth defect of the *atg6Δ* strain highlights a particularly strong requirement for the phosphatidylinositol 3-kinase complex in orchestrating autophagic degradation and vacuolar protein sorting. Collectively, these phenotypes highlight the critical importance of basal autophagy and vacuolar function in maintaining proteostasis and organelle quality. Furthermore, the reduced biomass accumulation under both carbon sources reinforces the essential role of autophagy-related pathways in supporting cellular growth and energy homeostasis in methylotrophic yeasts.

In light of our recent study^21^, which explored the regulation of peroxisomal dynamics and selective degradation mechanisms in *K. phaffii*, the present findings emphasize the importance of investigating how core autophagy genes coordinate broader cellular remodeling events. Future research should focus on identifying and characterizing additional molecular components that facilitate the vacuolar degradation of cytosolic proteins beyond canonical autophagic pathways. Such efforts, combining genetic, biochemical, and proteomic approaches, could provide a more comprehensive understanding of proteostasis networks and their regulatory hierarchies in methylotrophic yeasts.

## Conclusion

After shifting the cells of the methylotrophic yeast *K. phaffii* from methanol to glucose, the cytosolic enzymes Fld1 and Fdh1 undergo vacuolar degradation. Our findings demonstrate that the autophagy-related genes *ATG1*, *ATG6*, and *ATG15* are essential for this process, as their deletion impairs enzymatic turnover and compromises cellular adaptability during mentioned carbon source transitions. Together, these results highlight the pivotal role of the autophagic pathway in selective degradation of cytosolic enzymes of methanol metabolism in *K. phaffii* and provide a basis for future investigations into the molecular mechanisms underlying selective degradation of cytosolic proteins in yeasts.

## Materials and methods

### Strains and media

*K. phaffii* strains GS200 (*his4 arg4*)^22^, SMD1163 (*his4 pep4 prb1*)^10^, and *gss1Δ*^14^ were used in this study; all are derived from the same parental strain, GS115 (*his4*)^22^. Cells were cultivated in YPD medium (1% yeast extract, 2% peptone, 1% glucose) or in YNB (0.67% Yeast Nitrogen Base, Difco) mineral medium supplemented with methanol (0.5%), ethanol (1%), or glucose (2%) as carbon sources. For the growth of auxotrophic strains on mineral media, appropriate growth factors were added: amino acids L-histidine − 50 mg/L, L-arginine − 50 mg/L. All solid media contained 2% agar. *K. phaffii* strains were grown at 28 °C, 220 rpm. The *Escherichia coli* strain DH5α [Φ80d*lac*ZΔM15, *recA*1, *endA*1, *gyrA*96, *thi-*1, *hsdR*17(r^−^_K_ m^+^_K_), *supE*44, *relA*1, *deoR*, Δ(*lacZYA-argF*) U169] was used in experiments that required a bacterial host. DH5α was grown at 37 °C in LB medium as described^23^. Transformed *E. coli* cells were maintained in rich medium containing 100 mg/L of ampicillin or 50 mg/L of nourseothricin (NTC).

### Biochemical methods and analyses

Yeast cells were grown in YPD medium overnight, and afterwards transferred to the fresh YPD medium and further incubated for 6 h. Then cells were collected by centrifugation, washed twice with water, shifted to medium with 1% methanol for induction of methanol catabolism enzymes Fld1 and Fdh1 and incubated for 18–23 h. For initiation of target proteins degradation, cells were transferred to fresh medium containing 2% glucose at OD600 of 0.5. Cell biomass of yeast was determined with a Helios Gamma spectrophotometer (OD, 600 nm; cuvette, 10 mm) turbidimetrically with gravimetric calibration.

The activity of Fld1 and Fdh1 was measured as has been described elsewhere, with slight modifications^24^. To determine the specific activity of Fld1 and Fdh1, yeast cells were grown to mid-log phase on YNB and cell-free extracts were prepared by using glass beads. Protein concentration was determined by the Lowry method^25^ using bovine serum albumin as a standard. The activity of Fld1 was determined in the reaction mixture of the following composition (1 mL): 1 mM formaldehyde, 1 mM NAD^+^ and 2 mM glutathione in 100 mM phosphate buffer, pH 8.0. Fdh1 activity was evaluated in the following reaction mixture (1 mL): 200 mM NaCOOH and 2 mM NAD^+^ in 100 mM phosphate buffer, pH 7.5. The reaction was started by the addition of a cell-free extract at a concentration of 0.1 mg/mL to the mixture, and the kinetics of the change of extinction at a wavelength of 340 nm was determined. This wavelength corresponds to the peak absorption of nicotinamide cofactors. The Fld1 and Fdh1 activities were calculated according to the following equation: U/mL of enzyme =(OD340/min)/(6.22 × mg of enzyme/mL of reaction mixture), where OD 340/min is the change in extinction at a wavelength of 340 nm per unit time (min); 6.22 is the extinction coefficient of nicotinamide cofactors.

The Aox1 activity was assayed based on the rate of hydrogen peroxide formation in the reaction with methanol, as monitored by the peroxidative oxidation of o-dianisidine in the presence of horseradish peroxidase^26^. To visualize Aox1 activity in yeast patches, cells were pregrown on YNB medium supplemented with methanol and then replica-plated onto solid YNB medium containing glucose. After incubation for 8 h, Aox1 activity was visualized by overlaying the patches with an Aox1 reaction mixture containing a permeabilizing agent^27^.

Western blot analysis was carried out as described previously^28^ with anti-Green Fluorescent Protein (GFP) antibody. Protein samples for Western blot analysis were prepared at 0, 6, and 24 h of glucose adaptation. Samples for analysis were prepared using 12.5% trichloroacetic acid and boiling with β-mercaptoethanol^28^. Antigen–antibody complexes were detected by enhanced chemiluminescence.

### Estimation of yeast viability with phloxine B

The mutant strains were grown in YPD medium for 18 to 24 h at 28 ℃ with shaking at 220 rpm. The cells were washed twice with sterilized water and inoculated to the nitrogen starvation YNB medium (0.17% yeast nitrogen base without ammonium sulfate and amino acids and 2% glucose) to initiate general autophagy at 28 ℃ with shaking at 220 rpm. Phloxine B staining was used to assess yeast viability. After 24 h of cultivation under conditions of nitrogen starvation cells of yeast strains were stained with a phloxine B solution to a final concentration of 2 µg/mL. Phloxine B staining reflects the percentage of dead cells. A fluorescence microscope with a red filter was used to estimate stained dead cells. The GS200/FLD1-GFP or GS200/FDH1-GFP and SMD1163 were used as negative and positive control strains, respectively. For quantitative evaluation of dead cells, Phloxine B fluorescence was recorded using an excitation wavelength of 540 nm and an emission wavelength of 564 nm^29^. The fluorescence intensity was measured using a spectrofluorometer (RF-6000, Shimadzu, Kyoto, Japan). Fluorescence was measured in an aqueous suspension of yeast cells adjusted to OD600 of 0.2.

### Plasmids and strains construction

*FLD1*,* FDH1*,* ATG1*,* ATG6* and *ATG15* gene sequences were extracted from the *P. pastoris* genome database (https://mycocosm.jgi.doe.gov/Picpa1).

The DNA fragment containing *FLD1* promotor and *FLD1* ORF (2292 bp) was obtained by PCR using pair of primers Ko1016/Ko1017 (Sequences of all primers represented in Table S1) and the *K. phaffii* genomic DNA as a template. The vector harboring GFP gene (7518 bp) was amplified by PCR using primers Ko1019/Ko1018 and plasmid pPN6-GFP^13^, as a template. The resulting fragments were combined into single plasmid using Gibson Assembly. The resulted plasmid expressing *K. phaffii FLD1* fused to GFP was designed as pPN6-FLD1-GFP (Fig. S8a).

In the same manner *FDH1* gene with own promoter (2141 bp) was amplified by PCR using pair of primers Ko1021/Ko1022 and the *K. phaffii* genomic DNA as a template. The vector containing GFP gene (7497 bp) was amplified by PCR using primers Ko1023/Ko1024 and plasmid pPN6-GFP, as a template. The resulting fragments were combined into single plasmid using Gibson Assembly. The resulted plasmid expressing *K. phaffii FDH1* fused to GFP was designed as pPN6-FDH1-GFP (Fig. S8b).

*K. phaffii* GS200 and SMD1163 (strain defected in autophagy pathway) recipient strains were transformed by the plasmids pPN6-FLD1-GFP and pPN6-FDH1-GFP. The introduction of DNA into yeast cells was performed by electroporation. Prior to transformation, plasmids pPN6-FLD1-GFP and pPN6-FDH1-GFP were linearized with SalI and SacI, respectively. Selection of plasmid-containing transformants was carried out on a mineral medium without histidine. The presence of the corresponding plasmids in the genome of constructed strains GS200/FLD1-GFP, SMD1163/FLD1-GFP, GS200/FDH1-GFP and SMD1163/FDH1-GFP were confirmed by PCR (data not shown).

For construction of strains with the deletion of genes *ATG1*,* ATG6* and *ATG15* the corresponding deletion cassettes were constructed. 5’ parts of the genes *ATG1*,* ATG6* and *ATG15* were PCR-amplified from genomic DNA of *K. phaffii* using a pairs of primers Ko1268/Ko1269, Ko1276/Ko1277 and Ko1282/Ko1283, while 3’ parts - amplified with Ko1270/Ko1271, Ko1278/Ko1279 and Ko1284/Ko1285. Backbone plasmid pUC57 was amplified with primers Ko1264/Ko1265. Corresponding 5’ and 3’ fragments and pUC57 were Gibson Assembled generating intermediate plasmids. Then, selective marker *natNT2* conferring resistance to NTC was amplified from plasmid pFMN1-rosB-NAT^30^ with primers Ko1266/Ko1267, inserted into the BamHI and SalI sites of the intermediate vectors, and the resulting plasmids were designated as pATG1Δ, pATG6Δ and pATG15Δ (Fig. S9a, S10a, S11a).

PCR fragments containing deletion cassettes were amplified with plasmids pATG1Δ, pATG6Δ and pATG15Δ with primers Ko1268/Ko1271, Ko1276/Ko1279 and Ko1282/Ko1285, respectively. PCR fragments were transformed into GS200/FLD1-GFP and GS200/FDH1-GFP. Selected strains GS200/FLD1-GFP/*atg1Δ;* GS200/FLD1-GFP/*atg6Δ;* GS200/FLD1-GFP/*atg15Δ* and GS200/FDH1-GFP/*atg1Δ;* GS200/FDH1-GFP/*atg6Δ;* GS200/FDH1-GFP/*atg15Δ* were examined by PCR. Fragments with predicted size were amplified using pairs of primers Ko1272/Ko1273 (718 bp) and Ko1274/Ko1275 (708 bp) for GS200/FLD1-GFP/*atg1Δ* and GS200/FDH1-GFP/*atg1Δ* (Fig. S9b, S9c); Ko1280/Ko1273 (716 bp) and Ko1274/Ko1281 (750 bp) for GS200/FLD1-GFP/*atg6Δ* and GS200/FDH1-GFP/*atg6Δ* (Fig. S10b, S10c); Ko1286/Ko1273 (720 bp) and Ko1274/Ko1287 (650 bp) for GS200/FLD1-GFP/*atg15Δ* and GS200/FDH1-GFP/*atg15Δ* (Fig. S11b, S11c) homologous to the sequence of *natNT2* marker and regions outside from the 5’ and 3’ parts from the target gene ORF used for recombination, respectively.

### Microscopy

Images were taken using a fluorescence microscope (Axio Imager A1; Carl Zeiss MicroImaging, Jena, Germany) in conjunction with a monochrome digital camera (Axio Cam MRm; Carl Zeiss MicroImaging). AxioVision 4.5 (Carl Zeiss MicroImaging) and ImageJ software were used for photo processing.

### Fluorescent assay

For quantitative analysis of GFP-tagged Fld1 and Fdh1 in yeast cells, fluorescence intensity was measured using a spectrofluorometer (RF-6000; Shimadzu, Kyoto, Japan) with excitation and emission wavelengths set to 490 nm and 515 nm, respectively. Measurements were performed in aqueous suspensions of cells containing target-GFP fusion proteins, adjusted to an OD₆₀₀ of 0.2.

### Statistical analysis

All experiments were performed using at least three independent biological replicates. Data are presented as mean ± standard deviation (SD). Statistical analysis (two-tailed Student’s t-test) was performed using Excel software. A value of *p* < 0.05 was considered statistically significant.      

## Supplementary Information

Below is the link to the electronic supplementary material.

Supplementary Material 1

## Data Availability

All datasets supporting the conclusions of this study are available within the article and its supplementary material.
