# Supplementary material for: Autophagic pathway is responsible for selective degradation of cytosolic enzymes formaldehyde and formate dehydrogenases in the methylotrophic yeast Komagataella phaffii
Source: Sci Rep. 2026 Jun 1;16:24946. doi: 10.1038/s41598-026-54529-6 (PMC13462316; doi:10.1038/s41598-026-54529-6)
Supplement: Supplementary file 1 [file 41598_2026_54529_MOESM1_ESM.pdf]

Table S1 List of primers used in this study (restriction sites are underlined)

Primer name	Primer sequence 5'-3'
Ko1016	GAT TGG AGC TCA TTA ATC TCA CCT CAG TTT TTG AAT C
Ko1017	GCT CAC GTG CAT AGT AAT CAC AGC ACG AA
Ko1018	TTA CTA TGC ACG TGA GCA AGG GCG AGG AGC TG
Ko1019	AGA TTA ATG AGC TCC AAT CAA GCC CAA TAA CTG
Ko1021	ATT GGA GCT CAA ATG GCA GAA GGA TCA GCC TG
Ko1022	TCA CTG CGA CCT TTT TGT CAT TAC CAT AAG
Ko1023	CAA AAA GGT CGC AGT GAG CAA GGG CGA GGA GCT G
Ko1024	GCC ATT TGA GCT CCA ATC AAG CCC AAT AAC TG
Ko1264	CCC AAG CTT GGC GTA ATC ATG GTC ATA G
Ko1265	TAG TCT AGA TGC ATT CGC GAG GTA CC
Ko1266	CGC GGA TCC GAC ATG GAG GCC CAG AAT ACC CTC
Ko1267	GCG GTC GAC CCA GTA TAG CGA CCA GCA TTC ACA TAC
Ko1268	TTC GAG CTC GGT ACC TCG CGA ATG CAT CTA GAC TAG TGA GTC TCT TCC TCT ATA TTT CCT TTC
Ko1269	AGG AGG GTA TTC TGG GCC TCC ATG TCG GAT CCG CGC CAA TCT TTC TGT CAG GCA TTT GTG
Ko1270	TAT GTG AAT GCT GGT CGC TAT ACT GGG TCG ACC GCG AGC CAA GAA ACT CGG AAG TAA TTA C
Ko1271	GAA ACA GCT ATG ACC ATG ATT ACG CCA AGC TTG GGT GTG GGC AAA TGG TCG AAG AAT AAC
Ko1272	ACC CAC GAC TCT CTA AAC TAC TGG
Ko1273	CAG TCA CAT CAT GCC CCT GAG C
Ko1274	CCC AGA TGC GAA GTT AAG TGC G
Ko1275	GAC CTC GCT GTG GAA TAA ATC TG
Ko1276	TTC GAG CTC GGT ACC TCG CGA ATG CAT CTA GAC TAA CTG CAT TAC TTG CCA TCT TTC CTT ATG
Ko1277	AGG AGG GTA TTC TGG GCC TCC ATG TCG GAT CCG CGT GGA AAG GGA ATT AGT AAA TAG GGG AC
Ko1278	TAT GTG AAT GCT GGT CGC TAT ACT GGG TCG ACC GCG ATA ATT GCC TTC AGT AGT TCA ACG TG
Ko1279	GAA ACA GCT ATG ACC ATG ATT ACG CCA AGC TTG GGC TTC TCT CTT CTT TCT TTC CTC ATC TC
Ko1280	TTC AGC GTT GAC CCA ACT GTG TTC
Ko1281	CAC GGG CTT AGA CAT ATC ATC GTC
Ko1282	TTC GAG CTC GGT ACC TCG CGA ATG CAT CTA GAC TAA ACT AGC ATC GAT ACA GAT GAC TGG
Ko1283	AGG AGG GTA TTC TGG GCC TCC ATG TCG GAT CCG CGC ATC AGT GTG ATG TAA ACG GAT ATG AAG
Ko1284	TAT GTG AAT GCT GGT CGC TAT ACT GGG TCG ACC GCA CAG TTG AGA ATT CAC CAC AGC TTT TTG
Ko1285	GAA ACA GCT ATG ACC ATG ATT ACG CCA AGC TTG GGA AAC GGA AGT GTA AGT CTG CAT TGG
Ko1286	GGA CGA ACA GTT AGA GAT TTC AGA G
Ko1287	GTC GAA TTG ACC CCA GAG AAC AAC

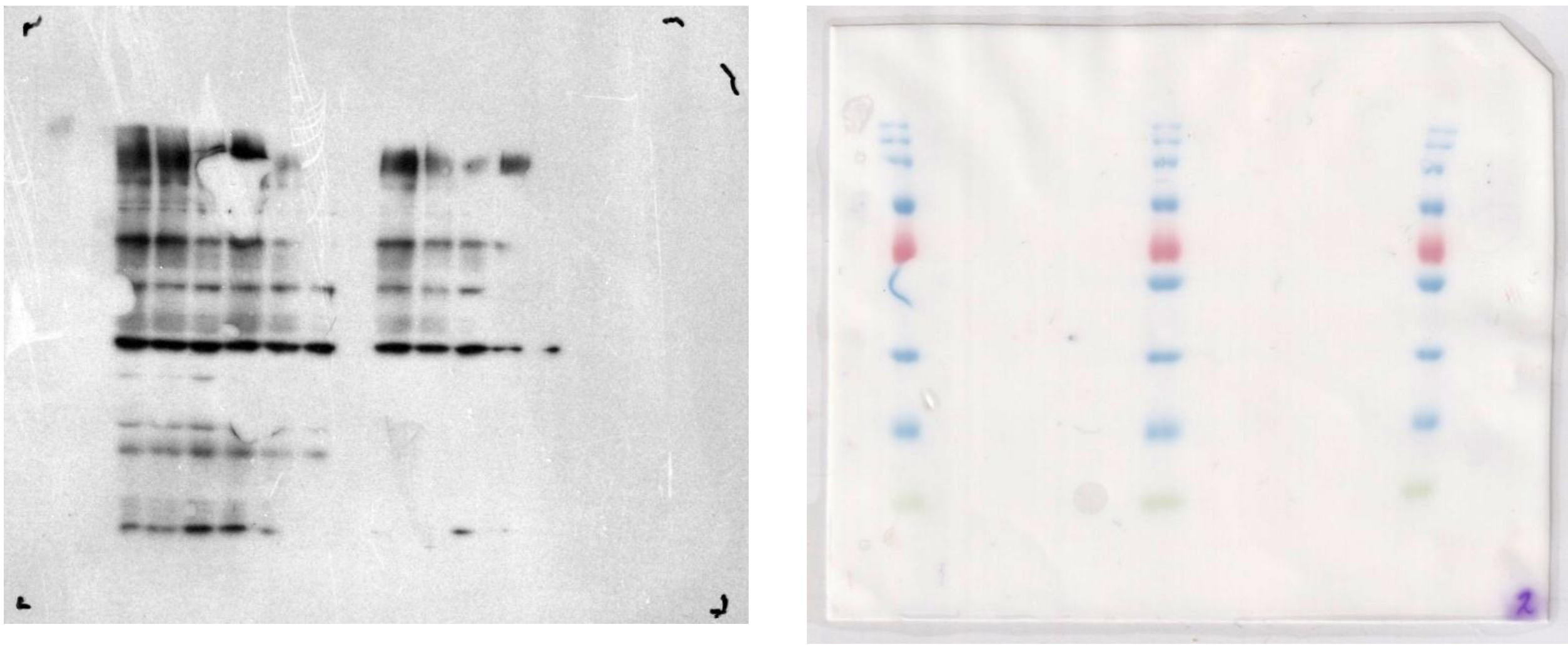

Fig. S1. Uncropped Western blot and corresponding membrane shown in **Fig.2(a)**. Broad Range Color Prestained Protein Standard (NEB) was used.

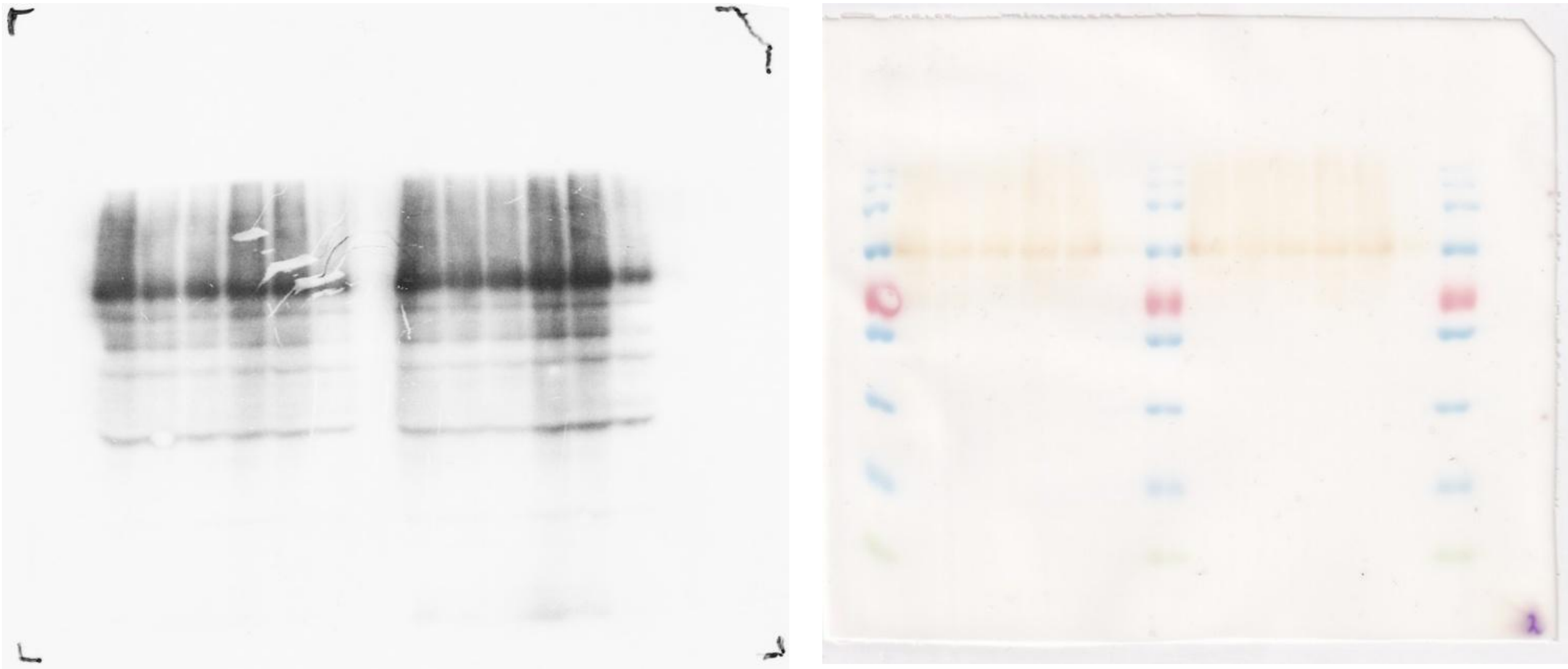

Fig. S2. Uncropped Western blot and corresponding membrane shown in **Fig.2(b)**. Broad Range Color Prestained Protein Standard (NEB) was used.

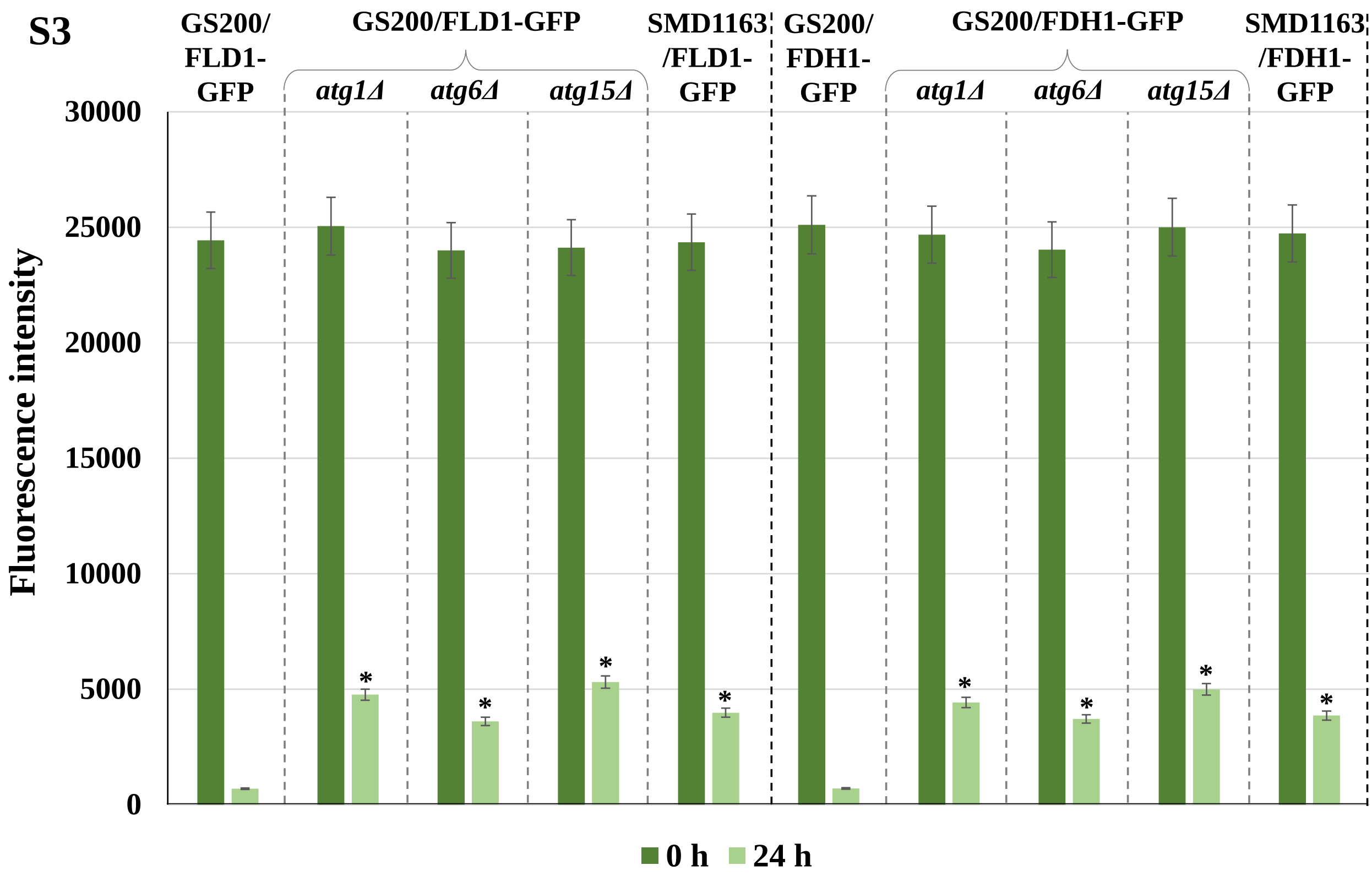

Fig. S3. Fluorescence intensity of GFP-tagged Fld1 and Fdh1 in *K. phaffii* strains GS200/FLD1-GFP, GS200/FLD1-GFP/atg1Δ, GS200/FLD1-GFP/atg6Δ, GS200/FLD1-GFP/atg15Δ, SMD1163/FLD1-GFP, GS200/FDH1-GFP, GS200/FDH1-GFP/atg1Δ, GS200/FDH1-GFP/atg6Δ, GS200/FDH1-GFP/atg15Δ, and SMD1163/FDH1-GFP. Cells were cultivated in methanol-containing medium (0 h) and subsequently transferred to glucose-containing medium and incubated for 24 h to induce degradation of Fld1 and Fdh1. Fluorescence intensity was measured using an RF-6000 spectrofluorometer (Shimadzu) with excitation and emission wavelengths of 540 and 564 nm, respectively. Data are presented as mean ± SD (n = 3 biological replicates). Differences relative to the GS200/FLD1-GFP or GS200/FDH1-GFP strains at the corresponding time point are indicated as $p < 0.05$ (*), Student's *t*-test.

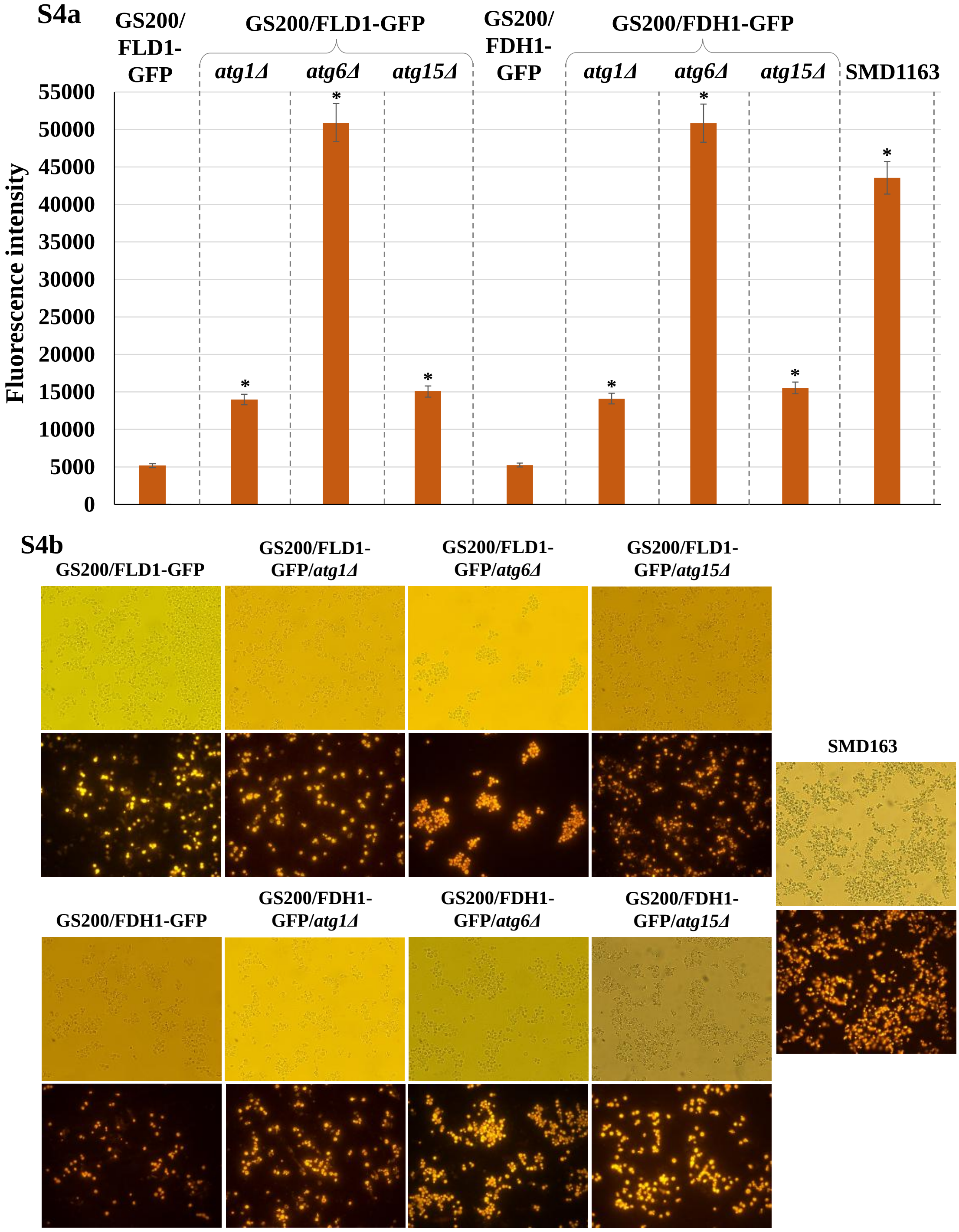

Fig. S4. (**a**) Fluorescence of Phloxine B–treated strains GS200/FLD1-GFP, GS200/FLD1-GFP/atg1Δ, GS200/FLD1-GFP/atg6Δ, GS200/FLD1-GFP/atg15Δ, GS200/FDH1-GFP, GS200/FDH1-GFP/atg1Δ, GS200/FDH1-GFP/atg6Δ, GS200/FDH1-GFP/atg15Δ, and SMD1163 measured 24 hours after transfer of cells to nitrogen-starvation YNB medium. Fluorescence intensity was recorded using an RF-6000 spectrofluorometer (Shimadzu) at excitation and emission wavelengths of 540 and 564 nm, respectively. Data are presented as mean ± SD (n = 3 biological replicates). Differences relative to the GS200/FLD1-GFP or GS200/FDH1-GFP strains at the corresponding time point are indicated as $p < 0.05$ (*), Student's *t*-test. (**b**) Fluorescence images of the GS200/FDH1-GFP, GS200/FLD1-GFP/*atg1Δ*, GS200/FLD1-GFP/*atg6Δ*, GS200/FLD1-GFP/*atg15Δ*, and GS200/FDH1-GFP, GS200/FDH1-GFP/*atg1Δ*, GS200/FDH1-GFP/*atg6Δ*, GS200/FDH1-GFP/*atg15Δ*, and SMD1163 strains stained with Phloxine B. Cells were pre-cultured in YPD liquid medium for 24 hours, washed twice with sterile water, and inoculated into nitrogen-starvation YNB medium at an initial OD600 of 0.5. Images were captured 24 hours after cultivation under nitrogen-starvation conditions.

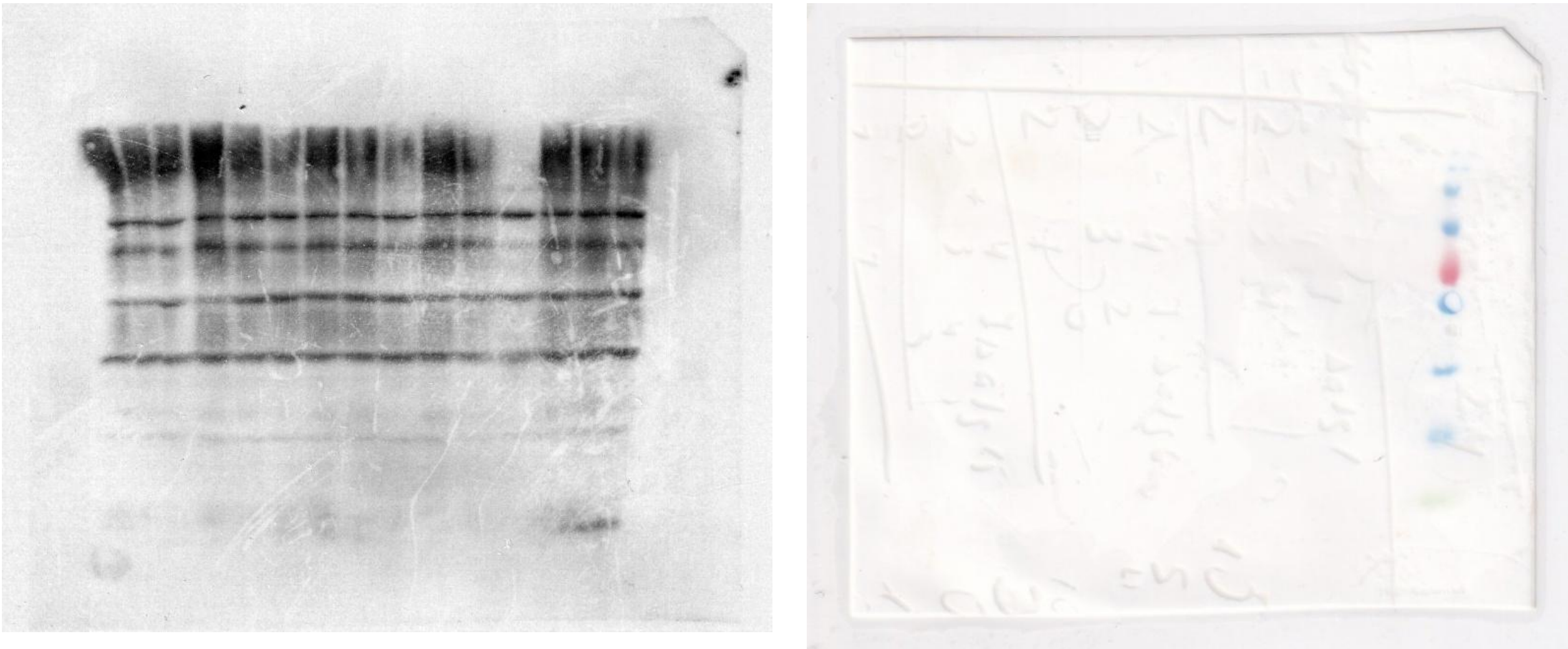

Fig. S5. Uncropped Western blot and corresponding membrane shown in **Fig.5(c)**. Broad Range Color Prestained Protein Standard (NEB) was used.

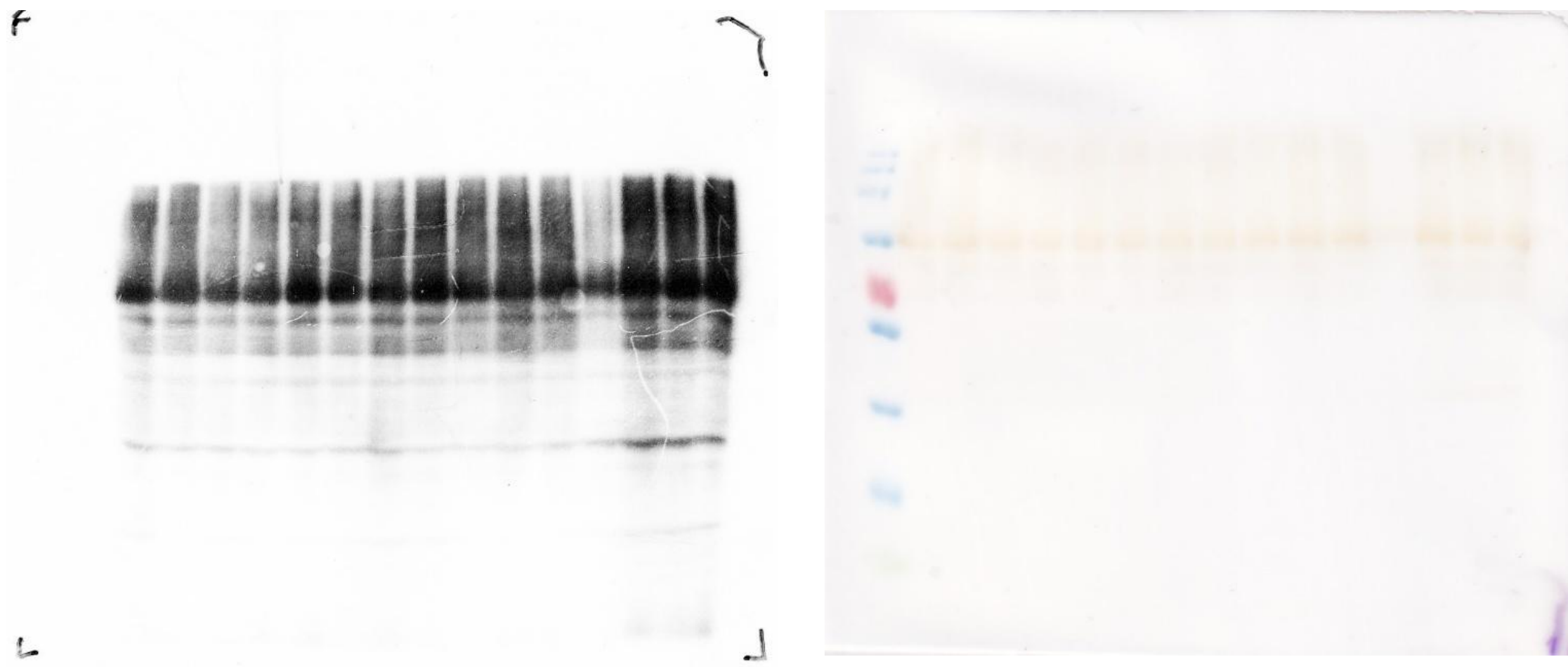

Fig. S6. Uncropped Western blot and corresponding membrane shown in **Fig.5(d)**. Broad Range Color Prestained Protein Standard (NEB) was used.

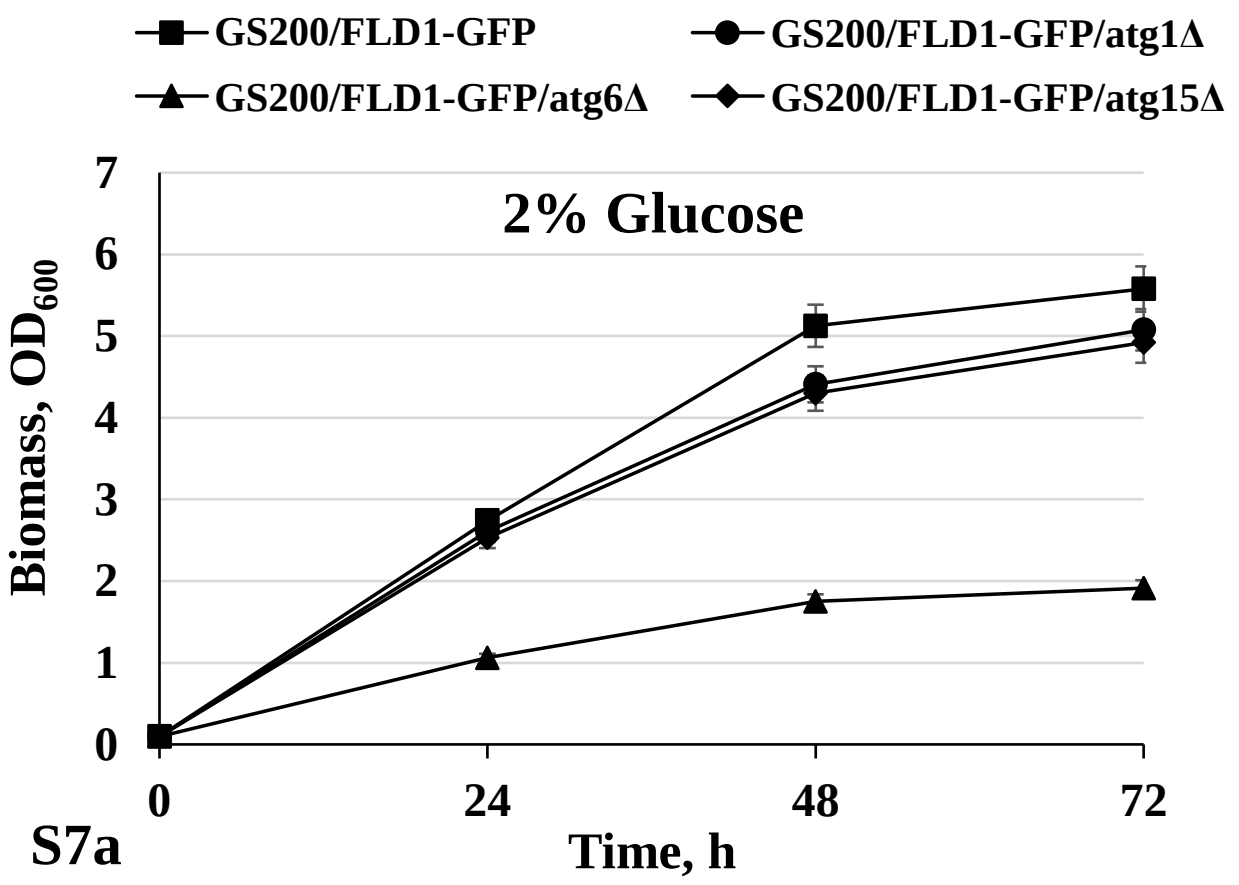

S7a

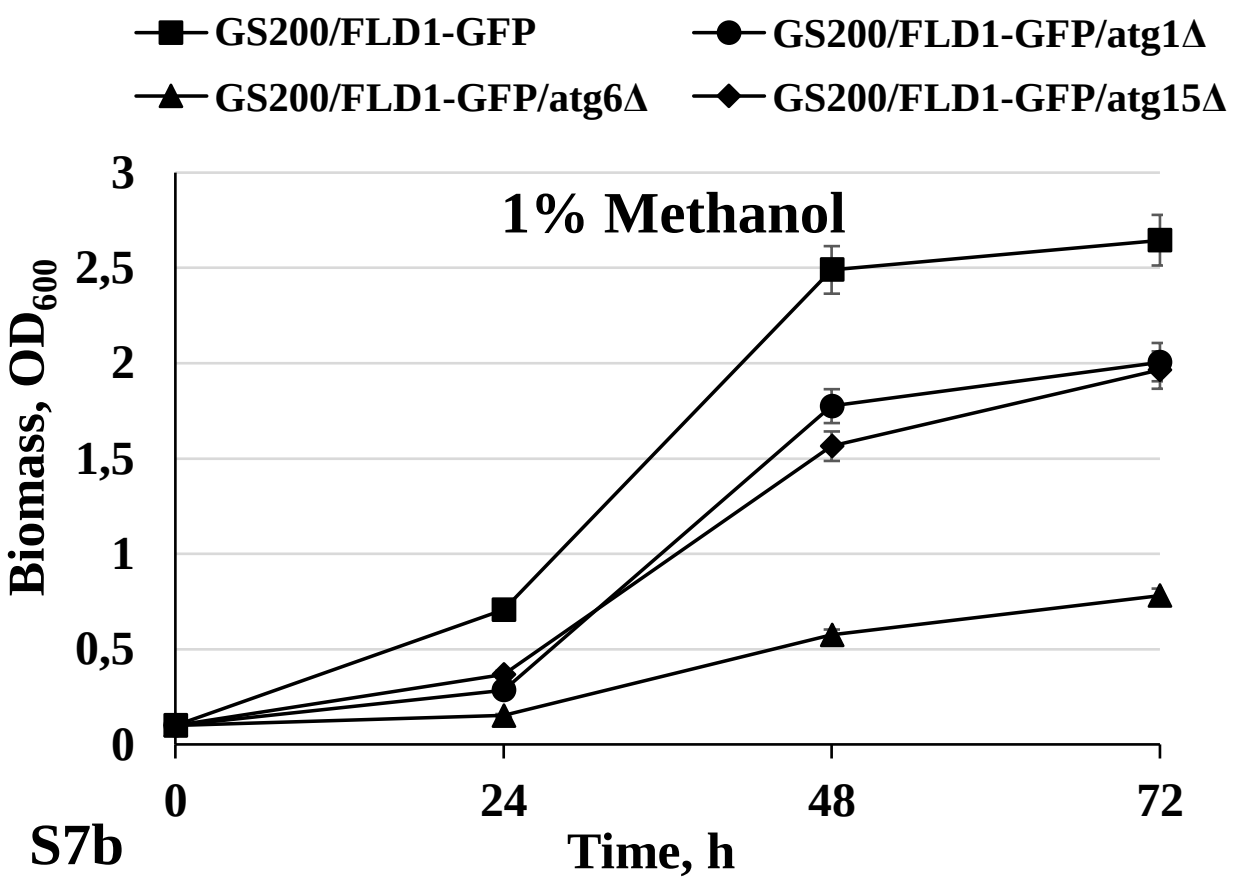

S7b

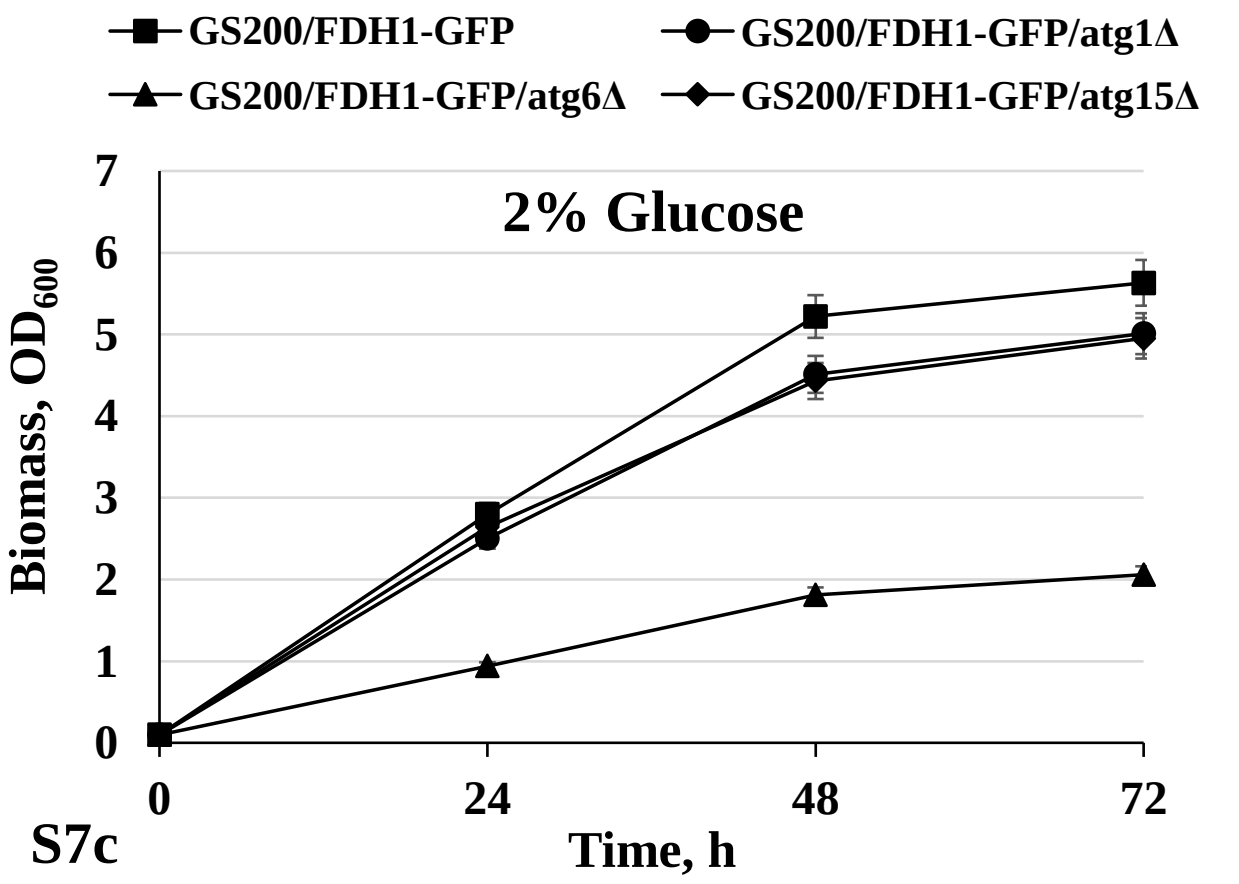

S7c

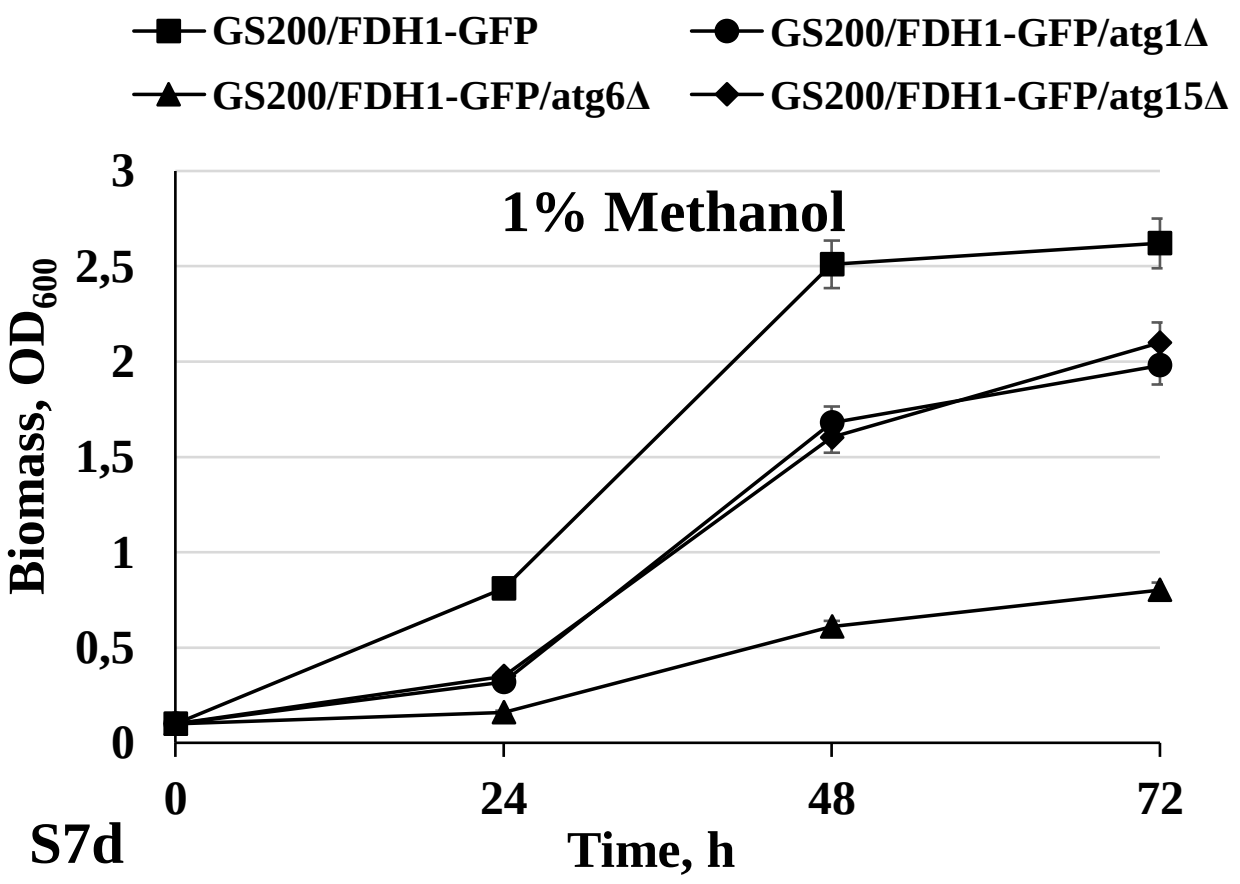

S7d

Fig. S7. Growth kinetics of GS200/FLD1-GFP/*atg1Δ*, GS200/FLD1-GFP/*atg6Δ*, GS200/FLD1-GFP/*atg15Δ*, GS200/FDH1-GFP/*atg1Δ*, GS200/FDH1-GFP/*atg6Δ*, GS200/FDH1-GFP/*atg15Δ* and parental strains GS200/FLD1-GFP, GS200/FDH1-GFP on different carbon sources. Cells were pre-grown in YPD liquid medium for 24 hours and then washed with water and inoculated 0.1 OD600 in YNB medium with 2% glucose (**a, c**) or 1% methanol (**b, d**), as carbon source.

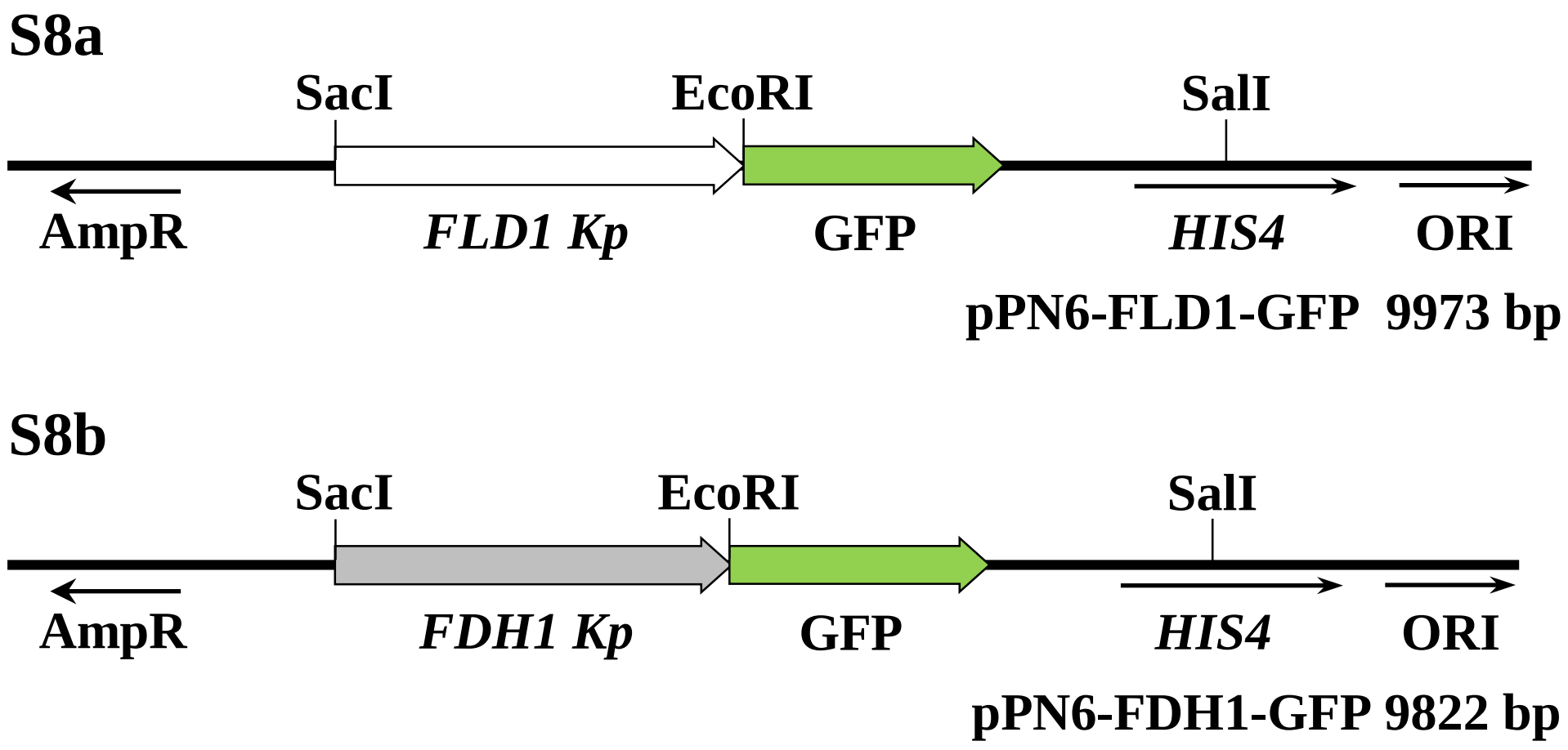

Fig. S8. Linear schemes of plasmids (**a**) pPN6-FLD1-GFP, (**b**) pPN6-FDH1-GFP. *FLD1* and *FDH1* genes of *K. phaffii* represented as open and grey arrow, espectively; GFP – green arrow; AmpR – ampicillin resistance gene; *HIS4* – encodes a histidine biosynthesis trifunctional protein; ORI - origin of replication. Restriction sites: SacI; EcoRI; SalI.

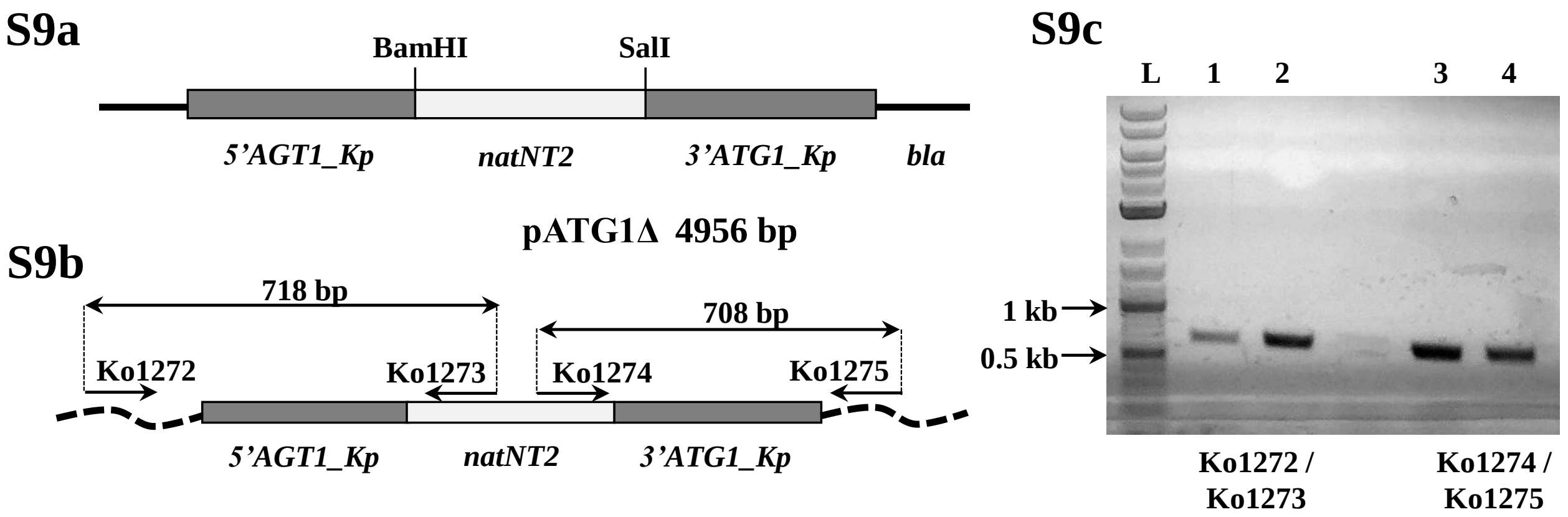

Fig. S9. (**a**) Scheme of plasmid pATG1Δ. (**b**) Scheme of *ATG1* deletion cassette integrated into the genome of *K. phaffii* with primers for PCR verification of the correct cassette integration. (**c**) PCR products with pairs of primers Ko1272/Ko1273 and Ko1274/Ko1275 and genomic DNA of GS200/FLD1-GFP/*atg1Δ* (lines 1, 3) and GS200/FDH1-GFP/*atg1Δ* (lines 2, 4) strain as a template confirming correct deletion of the gene *ATG1*; L – 1kb Plus DNA ladder.

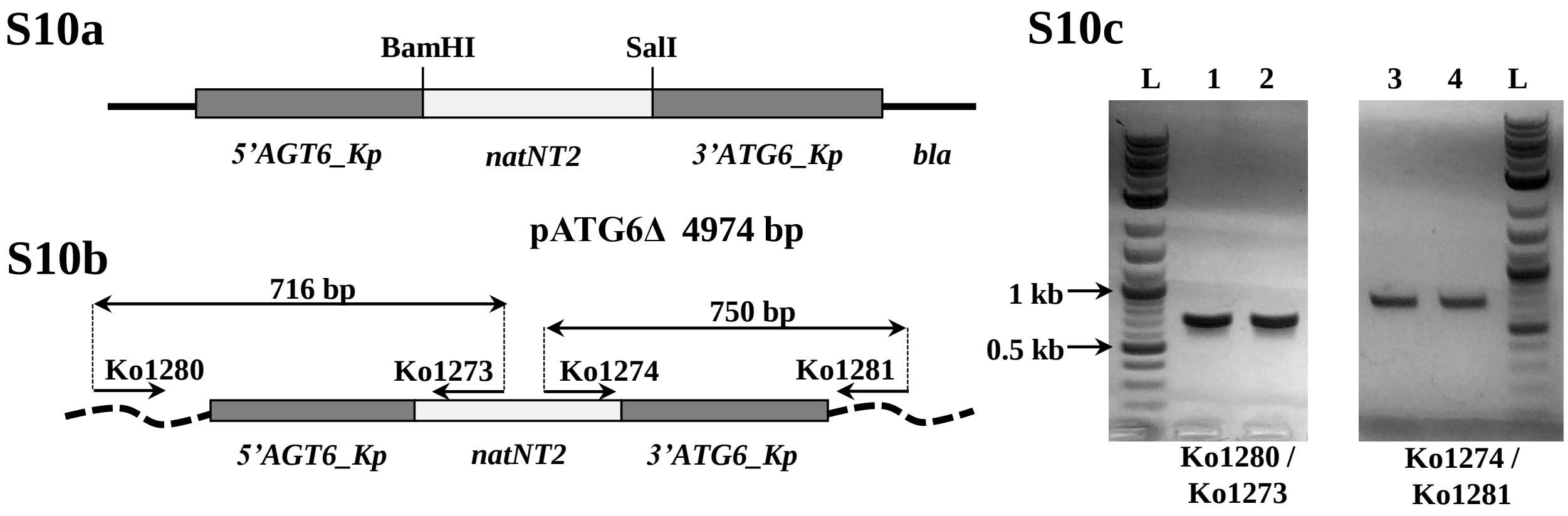

Fig. S10. (**a**) Scheme of plasmid pATG6Δ. (**b**) Scheme of *ATG6* deletion cassette integrated into the genome of *K. phaffii* with primers for PCR verification of the correct cassette integration. (**c**) PCR products with pairs of primers Ko1280/Ko1273 and Ko1274/Ko1281 and genomic DNA of GS200/FLD1-GFP/*atg6Δ* (lines 1, 3) and GS200/FDH1-GFP/*atg6Δ* (lines 2, 4) strain as a template confirming correct deletion of the gene *ATG6*; L – 1kb Plus DNA ladder.

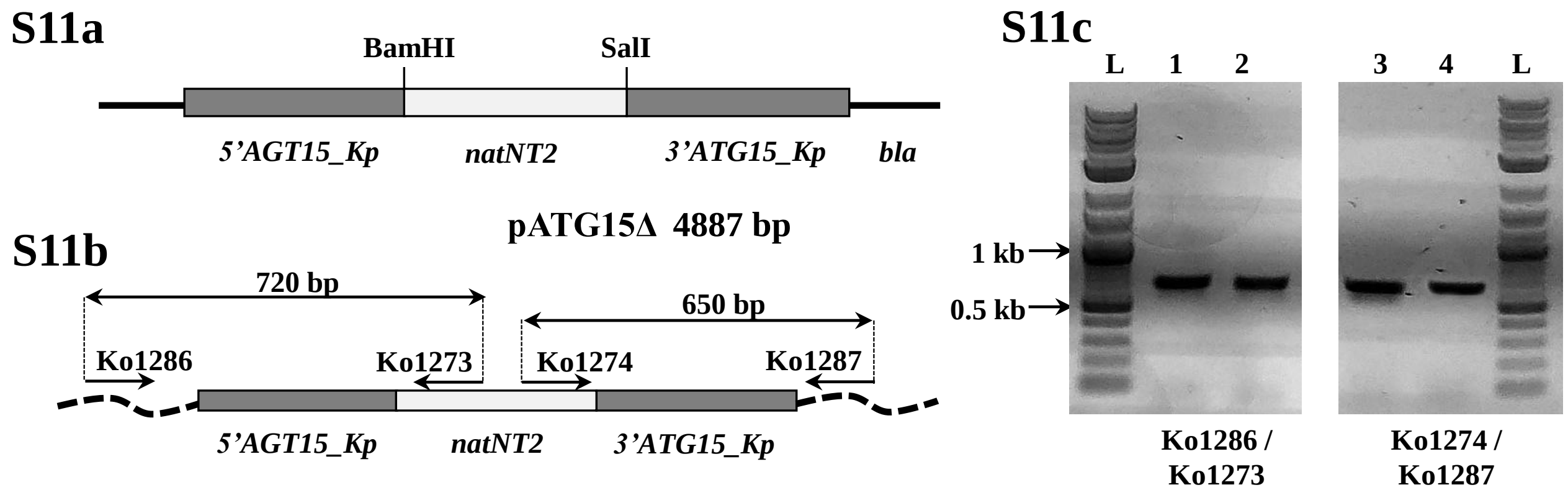

Fig. S11. (**a**) Scheme of plasmid pATG15Δ. (**b**) Scheme of *ATG15* deletion cassette integrated into the genome of *K. phaffii* with primers for PCR verification of the correct cassette integration. (**c**) PCR products with pairs of primers Ko1286/Ko1273 and Ko1274/Ko1287 and genomic DNA of GS200/FLD1-GFP/*atg15Δ* (lines 1, 3) and GS200/FDH1-GFP/*atg15Δ* (lines 2, 4) strain as a template confirming correct deletion of the gene *ATG15*; L – 1kb Plus DNA ladder.
